# Pharmacological Vitamin C Treatment Impedes the Growth of Endogenous Glutamine-Dependent Cancers by Targeting Glutamine Synthetase

**DOI:** 10.3389/fphar.2021.671902

**Published:** 2021-05-11

**Authors:** Yali Long, Jia Qiu, Bing Zhang, Peng He, Xinchong Shi, Qiao He, Zhifeng Chen, Wanqing Shen, Zhoulei Li, Xiangsong Zhang

**Affiliations:** Department of Nuclear Medicine, The First Affiliated Hospital of Sun Yat-Sen University, Guangzhou, China

**Keywords:** vitamin C, glutamine synthetase, redox stress, endogenous glutamine-dependent cancer, 13N-ammonia PET/CT

## Abstract

**Purpose:** Glutamine synthetase (GS) is the only currently known enzyme responsible for synthesizing endogenous glutamine (Gln). GS exerts a critical role in the oncogenesis of endogenous Gln-dependent cancers, making it an attractive target for anti-tumor therapies. A mixed-function oxidation system consisting of vitamin C (VC), oxygen, and trace metals can oxidize GS and promote its degradation. The current study aims to explore the effect of pharmacological VC treatment on GS.

**Methods:** Endogenous Gln-dependent cancer lines (breast cancer MCF7 and prostate cancer PC3) were selected to establish chronic Gln-deprived MCF7 and PC3 cell models. The expression of GS in parental and chronic Gln-deprived tumor cells exposed to VC treatment and control was determined by Western blot analysis. The anti-cancer effects of VC on parental and chronic Gln-deprived tumor cells were assessed by CCK-8 and annexin V-FITC/PI FACS assays. In addition, changes in cellular reactive oxygen species (ROS), glutathione (GSH) levels and NADPH/NADP + ratio were analyzed to explore the underlying mechanisms. Moreover, BALB/c nude mice xenografting with parental and chronic Gln-deprived prostate cancer cells were constructed to evaluate the *in vivo* therapeutic effect of VC. Finally, tumor 13N-ammonia uptake in mice bearing prostate cancer xenografts was analyzed following treatment with VC and the expression of GS in xenografts were detected by immunohistochemistry.

**Results:** Cells overexpressing GS were obtained by chronic Gln deprivation. We found that the cytotoxic effect of VC on cancer cells was positively correlated with the expression of GS. Additionally, VC treatment led to a significant increase in ROS production, as well as GSH depletion and NADPH/NADP + reduction. These changes could be reversed by the antioxidant N-acetyl-L-cysteine (NAC). Furthermore, pharmacological VC treatment exhibited a more significant therapeutic effect on xenografts of prostate cancer cells overexpressing GS, that could be well monitored by 13N-ammonia PET/CT imaging.

**Conclusion**: Our findings indicate that VC can kill cancer cells by targeting glutamine synthetase to induce oxidative stress. VC could be used as an anti-cancer treatment for endogenous glutamine-dependent cancers.

## Introduction

Vitamin C (VC) is an essential micronutrient for human beings, and serves as a cofactor for a large number of biosynthetic enzymes (Padayatty et al., 2003; Padayatty and Levine, 2016). Numerous pioneering studies have highlighted that pharmacological VC treatment exhibits selective toxicity to cancer cells both *in vivo* and *in vitro*. Additionally, early clinical trials have documented that intravenous VC treatment could relieve symptoms and improve survival in patients with advanced cancers (Cameron and Pauling, 1976; Cameron and Pauling, 1978; Chen et al., 2005; Chen et al., 2008; van der Reest and Gottlieb, 2016). Moreover, recent studies have shown that the anti-cancer effect of VC may be closely associated with its pro-oxidative function, which leads to increased levels of H_2_O_2_ and labile iron pool (LIP), consequently resulting in intracellular oxidative stress and induction of cell death (Yun et al., 2015; Schoenfeld et al., 2017; Cimmino et al., 2018). However, the biological functions of VC are highly diverse and can target multiple critical pathways in various cancers. The molecular mechanism underlying the cytotoxicity of VC remains to be established (Ngo et al., 2019).

The increased usage of glutamine (Gln) is a key feature of metabolic reprogramming in various types of cancers (Wise and Thompson, 2010; Ward and Thompson, 2012). In addition, Gln is the richest conditionally essential amino acid in plasma, accounting for as much as 20% of the total amino acid content. Moreover, it serves as a precursor for the synthesis of numerous amino acids, proteins, nucleotides, and other important biological molecules, in addition to being important in other processes such as supporting energy generation, modulating signaling pathways, and maintaining redox status (Stumvoll et al., 1999; DeBerardinis and Cheng, 2010; Hensley et al., 2013; Lyssiotis et al., 2013; Altman et al., 2016; Bott A. et al., 2019). However, due to highly fibrotic and poorly vascular diffusivity of tumor microenvironment, the ability of cells to uptake Gln from the tumor microenvironment is limited and depends on the supply of endogenous Gln. As the only known enzyme that catalyzes the synthesis of endogenous Gln from glutamate and ammonia in an adenosine triphosphate (ATP)-dependent pathway, glutamine synthetase (GS) has been reported to be generally overexpressed in breast, prostate and pancreatic cancers, where it maintains the proliferation and survival of cancer cells when the source of Gln is limited (Listrom et al., 1997; Pissimissis et al., 2009; Kung et al., 2011; Yuneva et al., 2012; Shi et al., 2014; Bott et al., 2015). In the endogenous Gln-dependent cancers, inhibition of GS expression is associated with a reduction in Gln and later leads to cell death. Therefore, targeting GS is a conceivable strategy against cancer metastasis and to improve the survival of patients with malignant tumors (Loeppen et al., 2002; Tardito et al., 2015; Yang et al., 2016; Bott A. J. et al., 2019).

Several studies have suggested that the GS enzyme is sensitive to oxidative stress. A mixed-function oxidation system comprising of O_2_, ascorbate, and trace metal, was previously demonstrated to be capable of modifying GS by altering one of its sixteen histidine residues/subunit, consequently causing the degradation of intracellular proteolytic enzymes (Fucci et al., 1983; Levine, 1983a; Levine, 1983b; Daggett, 1987). In this study, we investigate whether VC could selectively kill cancer cells by promoting GS proteolytic digestion. Our results showed that the cancer cells overexpressing GS were more sensitive to VC treatment, which reduces GS expression, and induces cell apoptosis and intracellular redox imbalance. Our findings indicate that VC has a positive therapeutic effect in endogenous Gln-dependent cancers through disrupting intracellular redox homeostasis by targeting GS.

## Materials and Methods

### Cells and Cell Culture

The endogenous Gln-dependent prostate cancer cell line PC3 and breast cancer cell line MCF7 were purchased from the Cell Bank of the Chinese Academy of Sciences (Shanghai, China). The obtained cells were cultured in RPMI 1640 (GIBICO, Grand Island, NY, United States) medium or DMEM medium (GIBICO, Grand Island, NY, United States) containing 10% FBS (GIBICO, Grand Island, NY, United States) and 1% penicillin/streptomycin (MRC, Changzhou, China) in a humidified incubator with 5% CO_2_ at 37°C. Chronic Gln-deprived PC3 and MCF7 cells were obtained by progressive Gln deprivation as previously reported (He et al., 2016): at each passage, half of the PC3 or MCF7 cells were seeded in a new culture medium with a Gln concentration half of that in the previous culture medium until cells grew at a Gln concentration of 0.06 mmol/L, and defined as 0.06PC3 and 0.06MCF7 cells.

### CCK-8 Assay

The CCK-8 assays were performed according to the manufacturer’s instructions (DOJINDO Laboratories, Kumamoto, Japan). Briefly, the cells were seeded in 96-well plates at a density of 1 × 10^4^ cells/well, and incubated with different doses (0, 0.5, 1, 2, 4, 8 mM) of VC (Alfa Aesar, Ward Hill, MA,United States) for 3, 6, and 9 h. Then, 100 μl CCK-8 solution was added to each well. After that the cells were incubated for an additional 1–2 h. Finally, the light absorbance values were recorded at 450 nm using a Multiskan FC apparatus (Thermo Fisher Scientific, Waltham, MA, United States) and cell viability was calculated.

### Flow Cytometry

Cells were incubated in 6-well plates at a density of 4 × 10^5^ cells/well with different doses (0, 0.5, 1, 2, 4, 8 mM) of VC at 37°C. After 16 h, the cells were harvested with 0.05% trypsin solution, rinsed twice with PBS and centrifuged at 1500 U/min for 5 min. Next, the cells were stained with fluorescein isothiocyanate-labelled Annexin V (BD Pharmingen, San Jose, CA, United States) and counterstained with propidium iodide (PI; BD Pharmingen, San Jose, CA, United States). Finally, the cells were resuspended in binding solution (BD Pharmingen) and analyzed using a flow cytometer (CytoFLEX S, Beckman Coulter, Fullerton, CA, United States).

### Western Blot Analysis

The drug-treated cells were washed, collected and lysed. The proteins were separated with 10% sodium dodecyl sulfate polyacrylamide gel electrophoresis (SDS-PAGE) and then transferred onto a polyvinylidene fluoride (PVDF) membrane. After blocking with 5% skimmed milk for 1 h, the membrane was incubated with primary rabbit anti-GS antibody (Abcam, Cambridge, United Kingdom) at 4°C overnight, while the mouse anti-β-tubulin was used as the internal reference protein. Next, the membrane was washed and incubated with the secondary antibody at room temperature for 1 h. The resulting blots were developed using a Pierce Fast Western Blot Kit (Thermo Fisher, Rockford, IL, United States) and exposed to film.

### ROS Assay

Cells were incubated in 6-well plates (at a density of 4 × 10^5^ cells/well) with or without 8 mM VC treatment for 2–4 h. After that, the cells were washed and incubated in serum-free medium containing 10 um/ml H2DCF-DA (GeneCopoeia, Rockville, MD, United States) for 30 min at 37°C in the dark. Next, the cells were collected after trypsinization and resuspended in 400 μl serum-free medium. Finally, the fluorescence-stained cells were measured using a flow cytometer (CytoFLEX S, Beckman Coulter, Fullerton, CA, United States).

### GSH Assay

The GSH assay was conducted based on the protocols provided by the GSH-Glo™ Glutathione Assay kit (Promega, Madison, WI, United States). Cells were seeded in 96-well white opaque plates at a density of 1 × 10^4^ cells/well and incubated with or without 8 mM VC for 3 h. After removing the drug from the wells and rinsing with PBS, the cells were incubated with 100 μl mixed GSH-Glo™ reagent for 30 min and 100 μl reconstituted luciferin detection reagent for another 15 min. Finally, intracellular luminescent signals were measured using a SPECTRAmaxM5 apparatus (Molecular Devices, Sunnyvale, CA, United States).

### The ratio of NADPH to NADP+

Cellular NADPH/NADP + ratio assay was performed according to the manufacturer’s instructions (Abbkine, Wuhan, China). Cells treated with 8 mM VC were collected after trypsinization. Next, the intracellular NADP+ and NADPH were extracted, placed on ice and added with 80 μl working solution quickly. Then, the solution was mixed gently and the light absorbance was measured at 565 nm using a Multiskan FC apparatus. After incubation for 30 min at room temperature, the light absorbance values were measured again and the NADPH/NADP + ratio was calculated as the instructions described.

### Xenograft Model in Nude Mice

BALB/c male nude mice (4–5 weeks old) were purchased from the Model Animal Research Center of Nanjing University (Nanjing, China). All animal experiments were approved by the Ethics Committee for Clinical Research and Animal Trials of the First Affiliated Hospital of Sun Yat-sen University and conformed to the Guide for the Care and Use of Laboratory Animals of the Ministry of Science and Technology of the People’s Republic of China. Firstly, 10 BALB/c male nude mice were randomly divided into two groups. Each nude mouse was subcutaneously injected with 2 × 10^6^ PC3 cells (right) and 0.06PC3 (left) cells in the two flank regions. Once the tumors reached a size of 50–80 mm^3^, the mice were respectively administered with saline or VC (4 g/kg, twice a day). Tumor sizes (length × width^2^ × 0.5) were measured once every 2 days using a Vernier caliper. After 16 days of treatment, the tumors were excised, weighed and analyzed by immunohistochemistry.

### Radiosynthesis of 13N-Ammonia

The 13N-ammonia was synthesized according to our published method (Xiangsong et al., 2006). We have done a purifucation step in our study. Radiochemical purity was obtained through IC-OH column to remove anions, and the radiochemical purity of 13N-ammonia was greater than 99%.

This part of the work and subsequent related operations strictly conformed to the Basic Standards for Protection Against Ionizing Radiation and for Safety of Radiation Sources of the People’s Republic of China (GB 18871–2002) and the safety management measures for laboratories of Sun Yat-sen University (NO. 2014–34).

### Positron Emission Tomography Imaging

13N-ammonia was administered *via* tail vein injection (100 μl) at an activity dose of 1 mCi per mouse 1 day before and 3 days after treatment initiation. CT scanning was started immediately after the injection, and then PET imaging was performed after 10 min using a micro-PET system (Inveon, SIEMENS, Germany). The emission protocol involved a 15 min static scan. Regions of interest were drawn around tumors and the contralateral normal tissues, and the tumor-to-background ratios (TBR) were calculated.

### Immunohistochemistry

Tumor specimens were obtained at the end of the treatment course. Cell proliferation were analyzed based on Ki-67 (Sevicebio, Palo Alto, CA, United States) staining. GS expression was analyzed after microPET/CT imaging. The immunohistochemical staining was carried out according to the previously described procedures (He et al., 2016; Zheng et al., 2020). Positive rate of Ki-67 was analyzed using Image-pro plus 6.0 (Media Cybernetics, United States). GS expression was quantified based on H-scores. The score is obtained by the following formula: 3 × the percentage of strongly stained nuclei + 2 × the percentage of moderately stained nuclei + 1 × the percentage of weakly stained nuclei. The range of H-scores is 0–300.

### Statistical Analysis

Comparisons between two groups were carried out using Student’s t test or analysis of variance (ANOVA) with Bonferroni post-hoc test. All analyses were performed in GraphPad Prism (GraphPad Software, Inc., La Jolla, CA, United States). Each experiment was repeated at least in triplicates, and the mean ± standard error of the mean (SEM) was calculated for each value. For all analyses, significance was determined at *p* < 0.05. *, **, *** or **** represent significant difference between drug exposure conditions.

## Results

### Chronic Glutamine Deprivation Significantly Increases Glutamine Synthetase Expression in MCF7 and PC3 Cells

**FIGURE 1:**
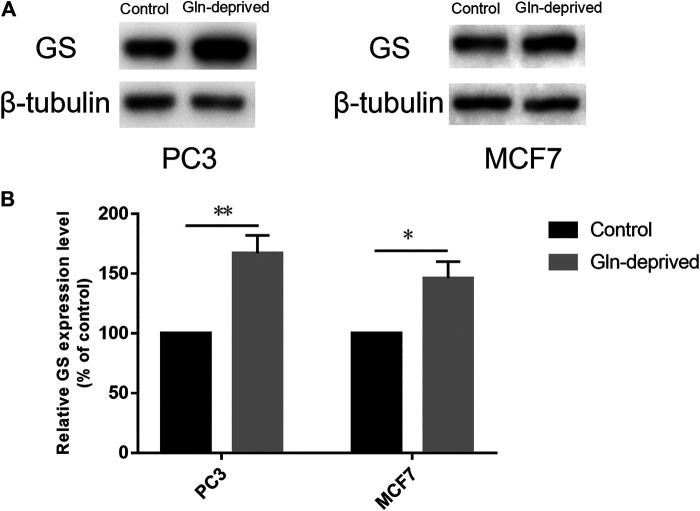
Chronic Gln deprivation augments the levels of GS in MCF7 and PC3 cells. **(A)** GS protein in cancer cells (MCF7、PC3、0.06MCF7、0.06PC3) analyzed by Western blotting. **(B)** Relative GS protein contents were evaluated with *β*-tubulin as a loading control. All data are presented as means ± SEM, *p < 0.05, **p < 0.01, *n* = 3. GS, glutamine synthetase.

Overexpression of GS has been documented in a wide array of cancers, wherein it maintains exogenous Gln independently and promotes the proliferative potential of cancer cells (Listrom et al., 1997; Yuneva et al., 2012; Pissimissis et al., 2009; Shi et al., 2014; Bott et al., 2015; Kung et al., 2011). We firstly applied Western blot analysis to determine the expression of GS protein in human prostate cancer cell line PC3 and breast cancer cell line MCF7. The results suggested that GS was expressed in both cell lines (Figure 1A). Previous studies have suggested that chronic Gln deprivation can augment the expression of GS both *in vitro* and *in vivo* (Collins et al., 1997; Kung et al., 2011; Tardito et al., 2015; Issaq et al., 2019). Based on this, in order to explore the anti-cancer effect of pharmacological VC on cancer cells with different GS protein expression levels, chronic Gln-deprived cell lines (0.06PC3, 0.06MCF7) were established and Western blot analysis was performed to examine their GS expression. It was found that elevated level of GS was seen in chronic Gln-deprived 0.06PC3 and 0.06MCF7 cells, as compared to their parental PC3 and MCF7 cells (Figures 1A,B).

**GRAPHICAL ABSTRACT:**
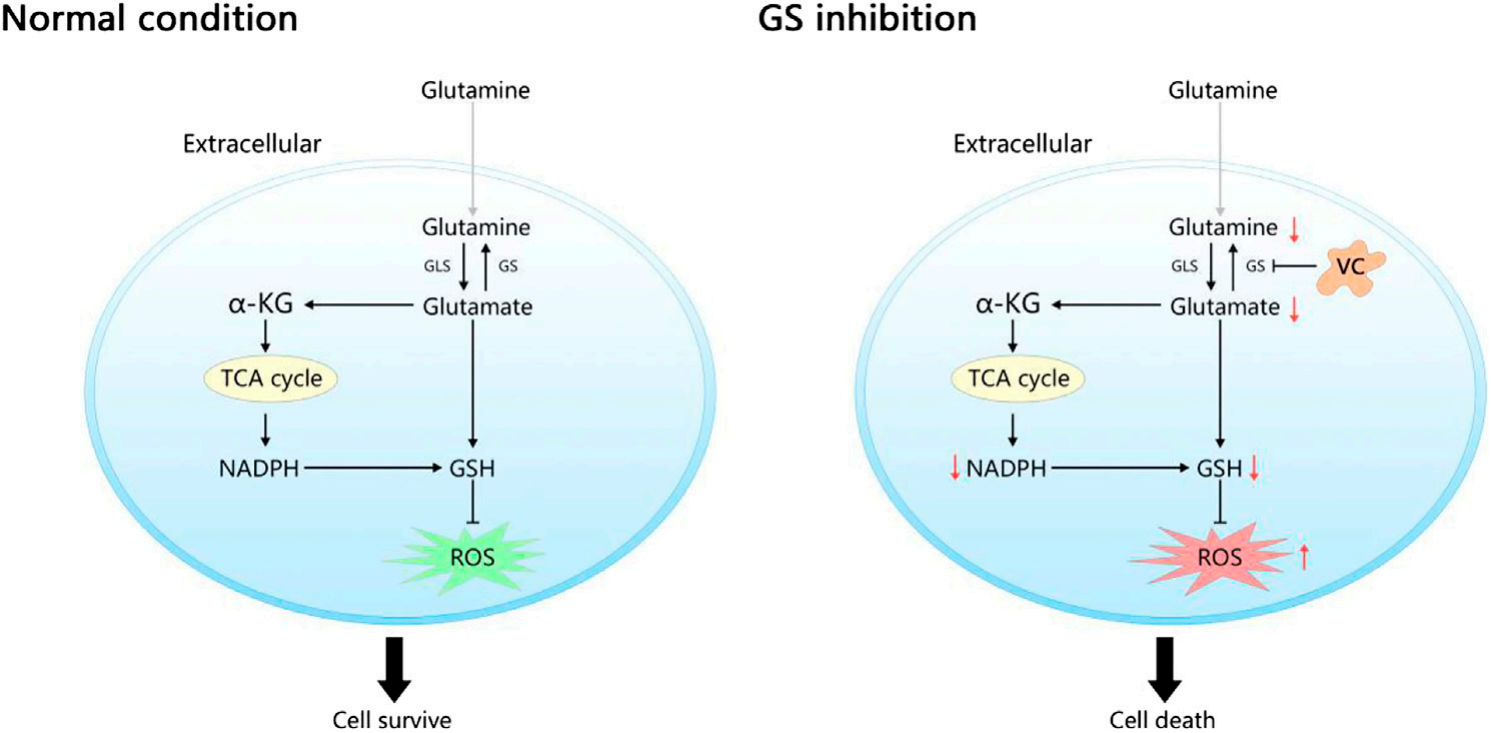
In normal condition, the endogenous Gln-dependent cancer cells generate Gln depending on glutamine synthetase (GS) for survival due to the limited ability of extracting circulating glutamine (Gln). Inhibition of GS by vitamin C (VC) limits Gln synthesis leads to a rapid depletion of intracellular nicotinamide adenine dinucleotide phosphate (NADPH) and glutathione (GSH) as well as an accumulation of reactive oxygen species (ROS), ultimately induces cell death.

### Cancer Cells Overexpressing Glutamine Synthetase are More Sensitive to Vitamin C Treatment

**FIGURE 2:**
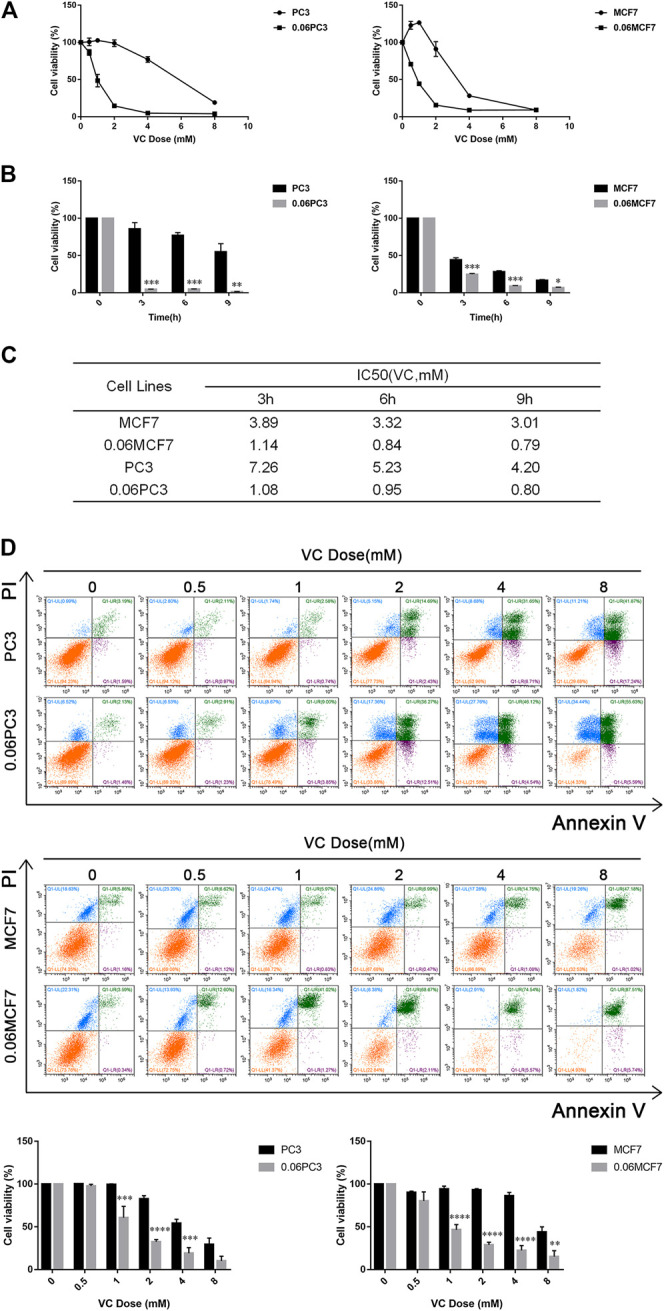
The cytotoxicity of VC increases in cancer cells overexpressed GS. **(A)** Cell viability evaluated by CCK-8 assay after treatment with different doses of VC for 6 h. **(B)** Cell viability evaluated by CCK-8 assay after treatment with VC (4 mM) for the indicated times. **(C)** IC50 of VC in cells evaluated by CCK-8 assay for the indicated times. **(D)** Cell apoptosis evaluated by annexin-V/PI assay after treatment with different doses of VC for 16 h. All data are presented as means ± SEM, *p < 0.05, **p < 0.01, ***p < 0.001, ****p < 0.0001, *n* = 3. VC, Vitamin C.

The expression of GS has been reported to be closely associated with progression and metastasis in numerous cancers (Son et al., 2013; Alberghina and Gaglio, 2014). Although several studies have demonstrated that a mixed-function oxidation system consists of O_2_, ascorbate, and trace metal may oxidize GS enzyme and consequently lead to degradation of the protein, the relationship between the selective toxicity of VC and the expression of GS protein remains unclear (Fucci et al., 1983; Levine, 1983a; Levine, 1983b; Daggett, 1987). Therefore, we established cell models of chronic Gln-deprivation using breast cancer MCF7 and prostate cancer PC3 cells. It was found that the cells with chronic Gln-deprivation, named 0.06MCF7 and 0.06PC3 cells had up-regulated expression of GS. The different response of chronic Gln-deprived tumor cells (0.06MCF7 and 0.06PC3) and their parental cells (MCF7 and PC3) upon VC treatment was tested. We found that all cells (MCF7, PC3 and 0.06MCF7, 0.06PC3) exhibited time- and dose-dependent inhibition of cell proliferation under VC treatment in CCK-8 assay (Figures 2A,B). We assessed the IC50 values of VC in these cells, and notably, the 0.06MCF7 and 0.06PC3 cells were found to be more sensitive to VC treatment. As shown in Figure 2C, for MCF7 and 0.06MCF7 cells, the IC50 values of VC at 3, 6 and 9 h were calculated to be 3.89, 3.32, 3.01 and 1.14, 0.84, 0.79 mM, respectively. As for PC3 and 0.06PC3 cells, the IC50 values of VC at 3, 6 and 9 h were calculated to be 7.26, 5.23, 4.20 mM and 1.08, 0.95, 0.80 mM, respectively. In addition, flow cytometric analysis revealed that pharmacological dose of VC conferred stronger cytotoxic effect on 0.06PC3 and 0.06MCF7 cells compared to PC3 and MCF7 cells, as reflected by an obvious increase in Annexin V/PI positive cells and a remarkable decrease in Annexin V/PI negative cells (Figure 2D). Taken together, these findings indicate that the cytotoxic effects of VC increased in cancer cells with high GS expression.

### Cancer Cells Overexpressing Glutamine Synthetase Show Augmented Protein Degradation Under Vitamin C Treatment

**FIGURE 3:**
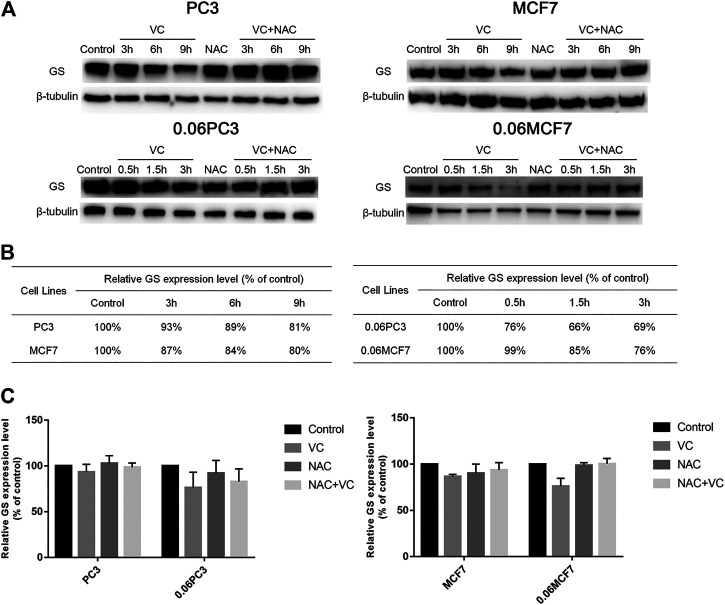
Cancer cells overexpressing GS show augmented protein degradation under VC treatment, which is reversed by NAC. **(A)** GS protein in cancer cells (MCF7、PC3、0.06MCF7、0.06PC3) analyzed by Western blotting under treatment with VC(4 mM), NAC(5 mM), VC(4 mM) + NAC(5 mM) for indicated times. **(B)** The relative GS contents of cancer cells (MCF7、0.06MCF7、PC3、0.06PC3) under treatment with VC(4 mM) for indicated times were calculated with *β*-tubulin as a loading control. **(C)** The relative expression of GS in cancer cells (MCF7、0.06MCF7、PC3、0.06PC3) under treatment with VC(4 mM), NAC(5 mM), VC(4 mM) + NAC(5 mM) for 3 h. All data are presented as means ± SEM, *n* = 3. VC, VitaminC; GS, glutaminesynthetase; NAC, N-acetyl-L-cysteine.

In order to clarify whether the cytotoxic effects of VC on tumor cells were related to the expression of GS, Western blot analysis was applied to quantify the changes in GS levels in tumor cells under 4 mM VC treatment at different times. As shown in Figure 3, the expression of GS decreased in a time-dependent manner in VC-treated cancer cells, as compared with the untreated cancer cells, and the GS degradation caused by VC was found to be reversed following pretreatment with the antioxidant N-acetyl-L-cysteine (NAC). In addition, the degradation level of GS was more significant in 0.06MCF7 and 0.06PC3 cells relative to MCF7 and PC3 cells. As shown in Figures 3B,C, after 3 h treatment with 4 mM VC, the expression of GS decreased to 76 and 69% in 0.06MCF7 and 0.06PC3 cells, respectively, in contrast to 87 and 93% in MCF7 and PC3 cells. When VC treatment went on for 9 h, the GS levels in MCF7 and PC3 cells decreased to 80 and 81%, respectively. These results together with the CCK-8 assay-based data suggested that cancer cells with up-regulated GS expression were more sensitive to VC treatment.

### Cancer Cells Overexpressing Glutamine Synthetase Show Augmented Redox Imbalance Under Vitamin C Treatment

**FIGURE 4:**
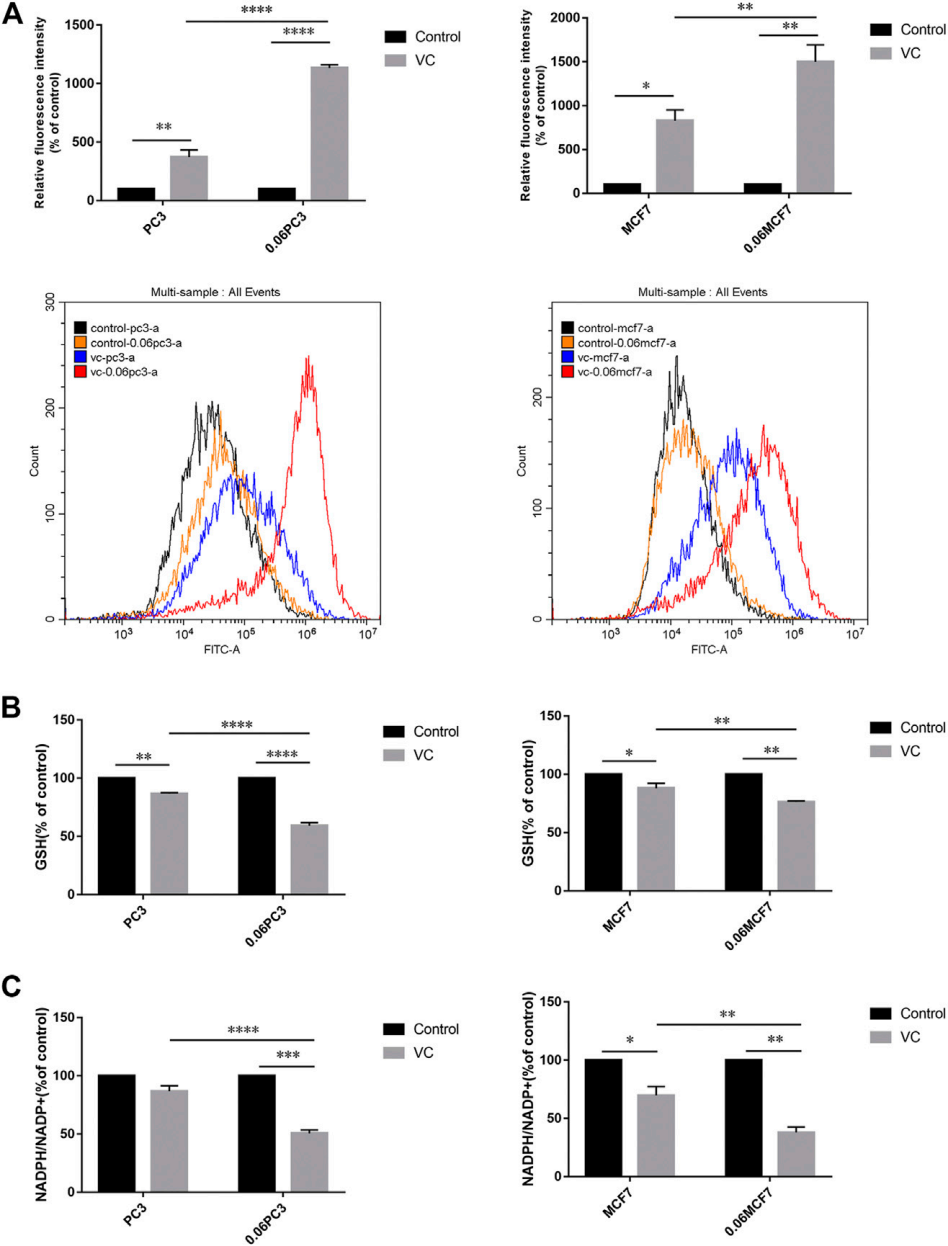
Cancer cells overexpressing GS show augmented redox imbalance under VC treatment. **(A)** Quantitative bar graphs of celllular ROS levels before and after treatment with VC (8mM, 3 h). **(B)** Quantitative bar graphs of celllular GSH levels before and after treatment with VC (8mM, 3 h). **(C)** Quantitative bar graphs of celllular NADPH/NADP + ratio before and after treatment with VC (8mM, 3 h). All data are presented as means ± SEM, *p < 0.05, **p < 0.01, ***p < 0.001, ****p < 0.0001, *n* = 3. VC, Vitamin C.

**FIGURE 5:**
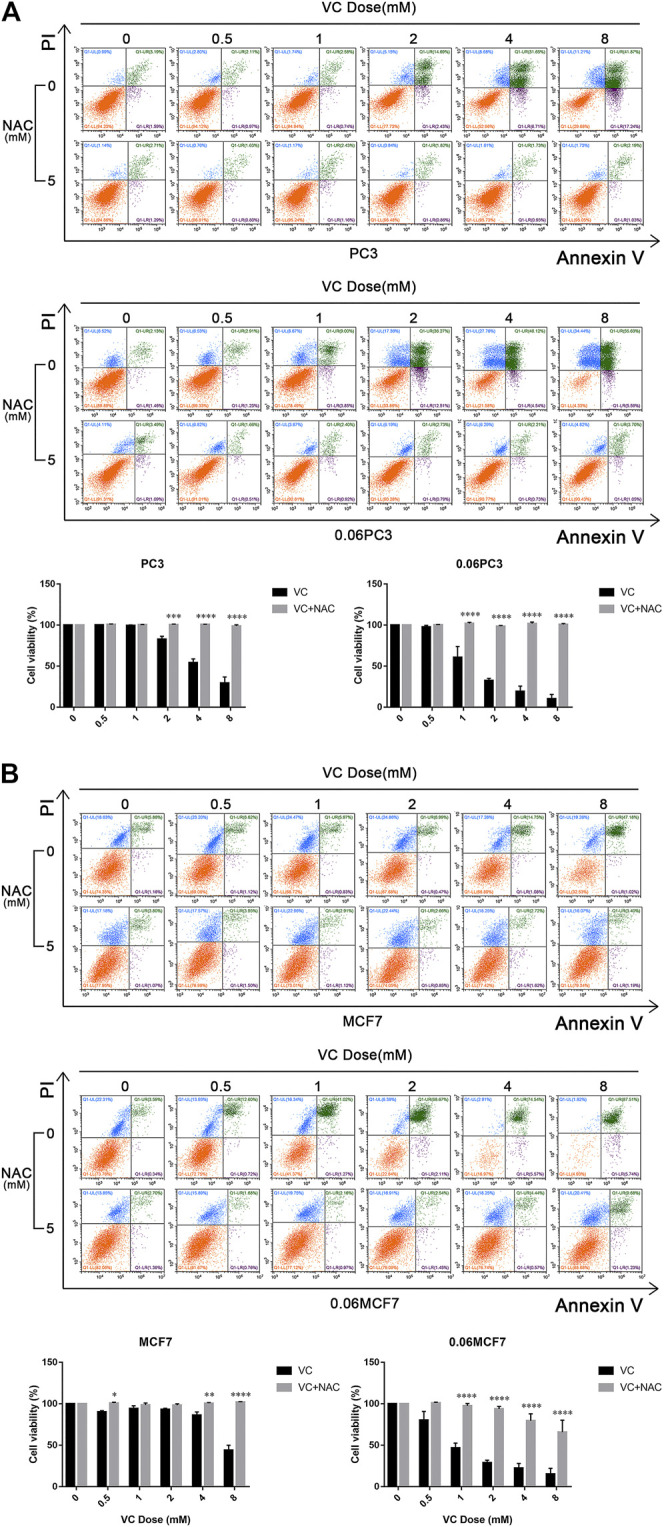
The cytotoxicity of VC reversed by NAC. Cell apoptosis evaluated by annexin-V/PI assay after treatment with VC(4 mM), NAC(5 mM), VC(4 mM) + NAC(5 mM) for indicated times. A and B. Pretreatment with 5 mM NAC for 1 h significantly prevented the cytotoxic effect induced by VC treatment. All data are presented as means ± SEM, *p < 0.05, **p < 0.01, ***p < 0.001, ****p < 0.0001, *n* = 3. VC, Vitamin C; NAC, N-acetyl-L-cysteine.

It was reported that Gln metabolism-sustained redox homeostasis is extremely critical, wherein inhibition of any component enzyme in this pathway can lead to an increased production of reactive oxygen species (ROS) and a decrease in reduced glutathione (GSH) (Lyssiotis et al., 2013). VC treatment induces toxicity in cancer cells due to disrupted redox balance, whereas the toxic mechanism and possible targets remain uncharacterized. We investigated whether VC treatment induces cancer cell apoptosis is associated with oxidative stress by targeting GS. Cellular ROS levels were detected by dichloro-fluorescein (DCF) staining after treatment with 8 mM VC for 3 h. As expected, the ROS production was found increased in all cells following VC treatment. Interestingly, the accumulation of ROS was more significant in 0.06MCF7 and 0.06PC3 cells, as compared with their parental MCF7 and PC3 cells. As shown in Figure 4A, the accumulation of ROS in 0.06MCF7 was about 1.8 times as that of MCF7 cells, whereas ROS accumulation in 0.06PC3 was 3.0 times of PC3 cells. Furthermore, we observed that changes in GSH depletion and NADPH/NADP + reduction were also obvious in 0.06MCF7 and 0.06PC3 cells, relative to MCF7 and PC3 cells (Figures 4B,C). Interestingly, the cell death and GS degradation could be reversed by pre-treatment with the antioxidant NAC (5 mM for 1 h) (Figure 3, Figure 5). These findings suggest that VC may lead to redox imbalance by targeting GS.

### Xenografts of Cells Overexpressing Glutamine Synthetase Show Slow Tumor Growth Under Vitamin C Treatment

**FIGURE 6:**
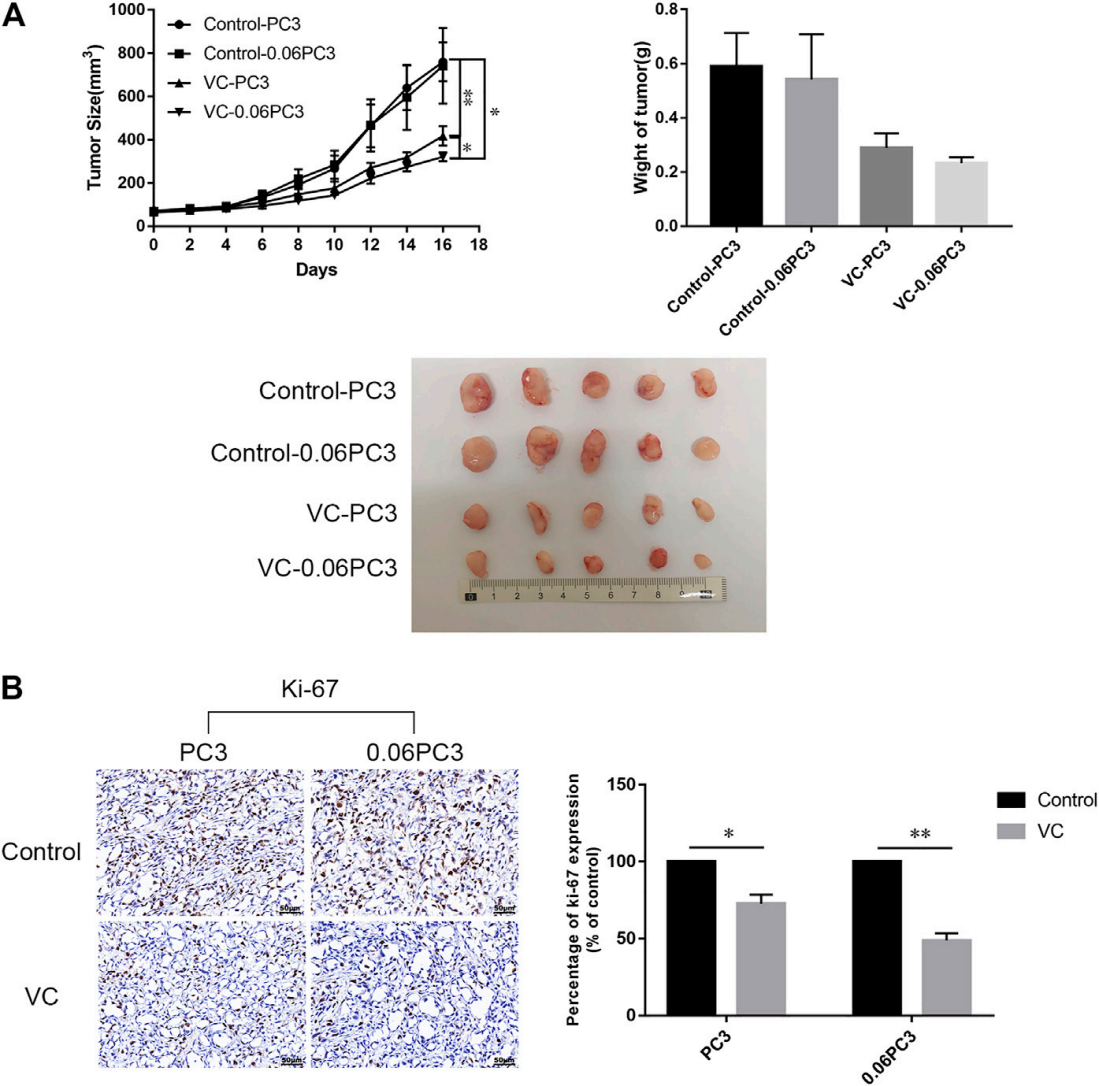
Xenografts of cells overexpressing GS show slow tumor growth under VC treatment. BALB/c nude mice (4–5 weeks old/male) were subcutaneously (s.c.) injected with 2 × 10^6^ PC3 cells. When the tumor sizes reached 50–80 mm^3^, ascorbate sodium (4 g/kg, twice a day) were intraperitoneally (i.p.) administered to the mice. After 16 days, all mice were euthanized and the tumors were analyzed. **(A)** Tumor volume and weight changed after treatment with VC. **(B)** Immunohistochemical analyses showed the expression of Ki-67 (proliferation marker) decreased in the VC treatment groups, with more obvious change in the 0.06PC3 group. All data are presented as means ± SEM, *p < 0.05, **p < 0.01, *n* = 3. VC, Vitamin C.

In order to determine the relationship between the *in vivo* anti-tumor activity of VC and GS expression in cancer cells, BALB/c male nude mice bearing xenograft tumors of PC3 and 0.06PC3 cells were intraperitoneally (i.p.) treated with 4 g/kg VC or saline (control) twice daily for 16 days. As shown in Figures 6A, VC treatment brought a remarkable reduction in tumor growth in tumors regardless of being derived from PC3(*p* = 0.0093) or 0.06PC3(*p* = 0.0433) cells, when compared to the control group. Moreover, VC conferred a more significant inhibition on tumor growth in the 0.06PC3 cell xenograft compared to PC3 cell xenograft (*p* = 0.0347). In addition, immunohistochemistry was employed to observe whether VC treatment could change the expression of proliferative index Ki-67 *in vivo*. It was found that the expression of Ki-67 decreased in response to VC treatment. Furthermore, the changes were found to be more significant in the 0.06PC3 cell xenograft model (Figure 6B). These findings confirm that pharmacological dose of VC has a more significant suppressive effect on prostate cancer xenograft of cells overexpressing GS by inhibiting cell proliferation *in vivo*.

### Tumor Xenografts Show a Significant Reduction in 13N-Ammonia Uptake Under Vitamin C Treatment

**FIGURE 7:**
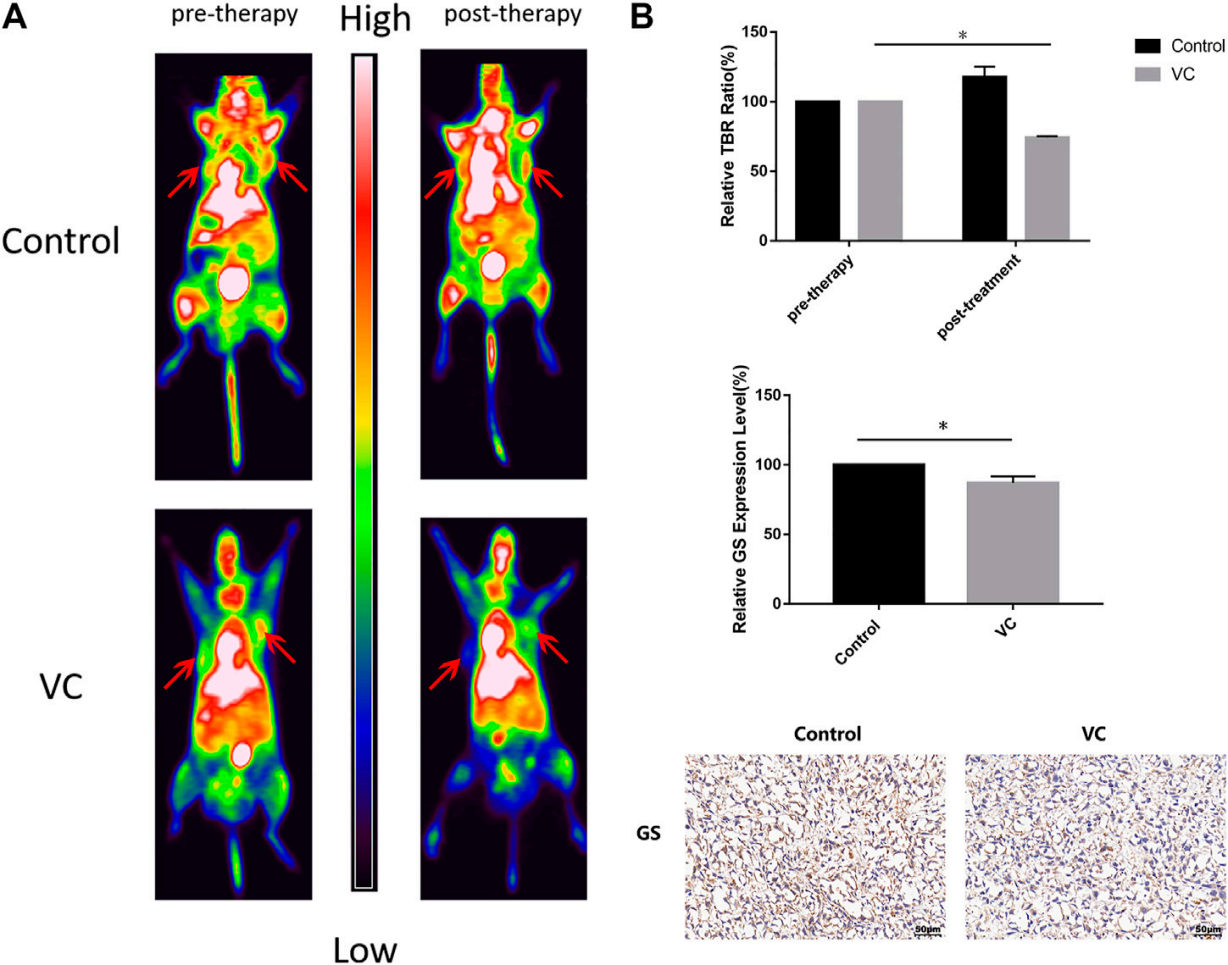
Functional 13N-ammonia PET/CT imaging of PC3 xenografts *in vivo* shows a significant reduction in 13N-ammonia uptake under VC treatment. **(A)** Tumor 13N-ammonia uptake in treated and control mice. 13N-ammonia PET/CT were conducted before (day 0) and 3 days after therapy initiation. Right panels, representative PET/CT scans showing change of tumor tracer uptake (red arrows). **(B)** Tumor-to-background ratio (TBR) was calculated and served as an indicator of tracer uptake. Mean TBR of 13N-ammonia PET/CT was reduced in VC treated mice compared to controls. Immunohistochemical analyses showed the expression of GS decreased in the VC treatment specimens. All data are presented as means ± SEM, *p < 0.05, *n* = 3. VC, Vitamin C; GS, glutamine synthetase.

Functional 13N-ammonia-PET/CT imaging was performed to evaluate the therapeutic response following VC treatment. BALB/c male nude mice bearing PC3 cell xenografts were intraperitoneally injected with 4 g/kg VC for 3 days when the volume of tumors had grown to 250–300 mm^3^, and the mice were intravenously injected with 1 mCi 13N-ammonia. As shown in Figure 7, 13N-ammonia-PET imaging revealed a visible reduction in 13N-ammonia uptake after 3 days of VC treatment compared with that before pretreatment, whereas the control group exhibited a slight increase in 13N-ammonia uptake. Subsequently, GS expression patterns in xenografts from the control group and VC treatment group were examined using immunohistochemistry, which demonstrated the weakened GS-positive staining in VC-treated sections (Figure 7B), suggesting that VC treatment could induce GS degradation. This result indicated that 13N-ammonia PET/CT imaging could be a useful tool in monitoring the therapeutic response.

## Discussion

Cancers are complex diseases involving numerous common signaling pathways and molecular metabolic alterations (Shenoy et al., 2018; Su et al., 2019). There have been a surge in the number of reports stating that some cancer cell proliferation relies on the increased consumption of Gln to support cell growth and maintain redox homeostasis (Hanahan and Weinberg, 2000; DeBerardinis et al., 2008; Gaglio et al., 2011). GS and the glutaminase (GLS) are commonly found enzymes that participate in Gln metabolism. GS possesses the ability to catalyze endogenous Gln production from glutamate and ammonia, which is the reverse reaction of glutaminolysis catalyzed by GLS. It was speculated that mutual suppressive action may exist between these two enzymes, that avoid their simultaneous up-regulation (Kung et al., 2011; Yuneva et al., 2012). Targeting GLS has been explored and documented to be beneficial for cancer therapy. However, some studies have found that inhibiting GLS does not always bring about desired results, especially in cancers with poor vascularization and limited ability of extracting circulating Gln, where they depend on the increased expression of GS to synthesize *de novo* Gln autonomously (Tardito et al., 2015; Bott A. J. et al., 2019). In addition, some other reports have indicated that many human cancers exhibit markedly elevated levels of GS, wherein GS enables cell growth independent on extracellular Gln. Silencing GS or using GS inhibitors can considerably suppress cell proliferation and tumor growth in preclinical models. However, there is no safe and specific drug targeting GS approved for clinical trials (Collins et al., 1997; Brusilow and Peters, 2017). In the current study, we found that 0.06PC3 and 0.06 MCF7 cells, that were derived from progressive Gln deprivation in PC3 and MCF7 cells, showed GS up-regulation and could grow normally in low-Gln medium. Moreover, the addition of VC could effectively inhibit cell proliferation and lead to GS degradation, which was more obvious in 0.06PC3 and 0.06 MCF7 cells with overexpression of GS, indicating that VC can kill cancer cells by targeting GS, especially in cells depending on GS expression. This study confirmed that Gln deprivation could up-regulate the expression of GS, which has been demonstrated and is one of the most commonly applied method for the regulation of GS expression (Collins et al., 1997; Tardito et al., 2015; He et al., 2016).

In the early 1970s, Cameron and Rotman first proposed that high-dose VC might confer anti-cancer property. Later on, numerous researches have also reported that VC exhibits selectively toxic effects on various types of cancer cells and holds immense application potential (Cameron and Pauling, 1976; Cameron and Pauling, 1978; van der Reest and Gottlieb, 2016; Shenoy et al., 2018). With further exploration on the role of VC, newly found evidences revealed that the selective anti-cancer toxicity of pharmacological VC treatment was regulated by the production of ascorbic acid radical and H_2_O_2_ in the extracellular fluid, a process driven by the depletion of NADPH and GSH (Chen et al., 2005; Yun et al., 2015; Schoenfeld et al., 2017). However, the targets and pathways of VC-mediated cell apoptosis are multiple, while the therapeutic effect of VC varies among different cancers. Notably, studies have reported that a system comprising of O_2_, VC and trace meta is capable of inducing GS degradation (Fucci et al., 1983; Levine, 1983a; Levine, 1983b; Daggett, 1987). In addition, it is also known that sustaining protein homeostasis is critical for cell growth and the balance between protein synthesis and degradation must be discreetly regulated to maintain sound functioning of the system. As a result, highly selected strategies aimed at down-regulation of targeted proteins have become increasingly popular in therapeutic anti-cancer applications (Luh et al., 2020). In the current study, we demonstrated that VC could induce cell death *in vitro* and inhibit tumor growth *in vivo*. Additionally, our findings revealed that the cancer cells overexpressed GS were significantly more sensitive to VC treatment. GS is the only identified enzyme that can drive the process of endogenous Gln biosynthesis. As we know, a critical function of Gln metabolism is to produce reducing equivalents in the form of NADPH, which may sustain redox balance and support cell survival (Shenoy et al., 2018; Su et al., 2019). Based on this, we investigated the effects of VC on cellular homeostasis to elucidate its potential anti-tumor mechanisms. We found that VC treatment could lead to a cellular redox imbalance *via* several aspects that include accumulation of ROS, depletion of GSH and reduction in the NADPH/NADP + ratio. Moreover, the changes in redox imbalance were positively associated with cell death as well as GS degradation, that were more obvious in GS overexpressing cells. Furthermore, the above effects altered by VC treatment could be rescued through media supplementation with the antioxidant NAC. Collectively, these data indicate that VC exerts its anti-cancer effects by inducing GS degradation, which could be a novel approach to target malignancies. It holds a tremendous application potential in some specific cancers that depend on the high expression of GS to produce endogenous Gln.

GS upregulation is prevalent in multiple human cancers, with significant differences in expression patterns between tumors. Therefore, evaluation of GS expression profiles in tumors may be necessary for predicting therapeutic responses. Previous studies have reported that 13N-ammonia serves as a useful PET tracer in several types of cancers, including malignant lymphomas, brain tumors and prostate cancers (Shi et al., 2013a; Shi et al., 2013b; Shi et al., 2015). Recently, Shi *et al.* associated the accumulation of 13N-ammonia in prostate cancers with GS expression levels, while He *et al.* demonstrated that 13N-ammonia functions as a potential tracer for the evaluation of GS as it can be trapped by cancer cells through the synthesis of *de novo* Gln (Shi et al., 2014). In the current study, we established prostate cancer xenografts through subcutaneous injection of PC3 cells into nude mice, treated mice with VC for 3 days when tumor volume reached about 250–300 mm^3^ and monitored the therapeutic effects of VC using 13N-ammonia-PET/CT imaging. Our findings illustrated that 13N-ammonia-PET/CT imaging could accurately identify the location of xenografts, and was suitable for monitoring the therapeutic response of VC. Furthermore, we observed that VC treatment precipitated a visible reduction in 13N-ammonia accumulation in prostate cancer xenografts. Moreover, xenografts derived from VC-treated nude mice presented with lower GS expression levels relative to saline-treated mice. These findings indicate that 13N-ammonia PET/CT imaging is useful for the evaluation of GS expression, and suitable for monitoring the therapeutic effects of VC treatment in patients with high GS expression.

Taken together, our data indicate that VC treatment induces redox stress by targeting GS in tumor cells. This study suggests that VC possesses specific anti-tumor property in endogenous Gln-dependent cancers that rely on GS to promote its growth and survival. Furthermore, the application of 13N-ammonia PET/CT imaging could be useful for monitoring and predicting the therapeutic response in cancers overexpressing GS.

## Data Availability

The original contributions presented in the study are included in the article/Supplementary Material, further inquiries can be directed to the corresponding authors.

## Abbreviations

VC, vitamin C; GS, glutamine synthetase; NAC, N-acetyl-L-cysteine; Gln, glutamine; NADPH, nicotinamide adenine dinucleotide phosphate; GSH, glutathione; ROS, reactive oxygen species; LIP, labile iron pool; ATP, adenosine triphosphate; DCF, dichloro-fluorescein; PET/CT, positron emission tomography/computed tomography.
