# Supplementary figures and images for: Pharmacological Vitamin C Treatment Impedes the Growth of Endogenous Glutamine-Dependent Cancers by Targeting Glutamine Synthetase
Source: Front Pharmacol. 2021 May 11;12:671902. doi: 10.3389/fphar.2021.671902 (PMC8150514; doi:10.3389/fphar.2021.671902)

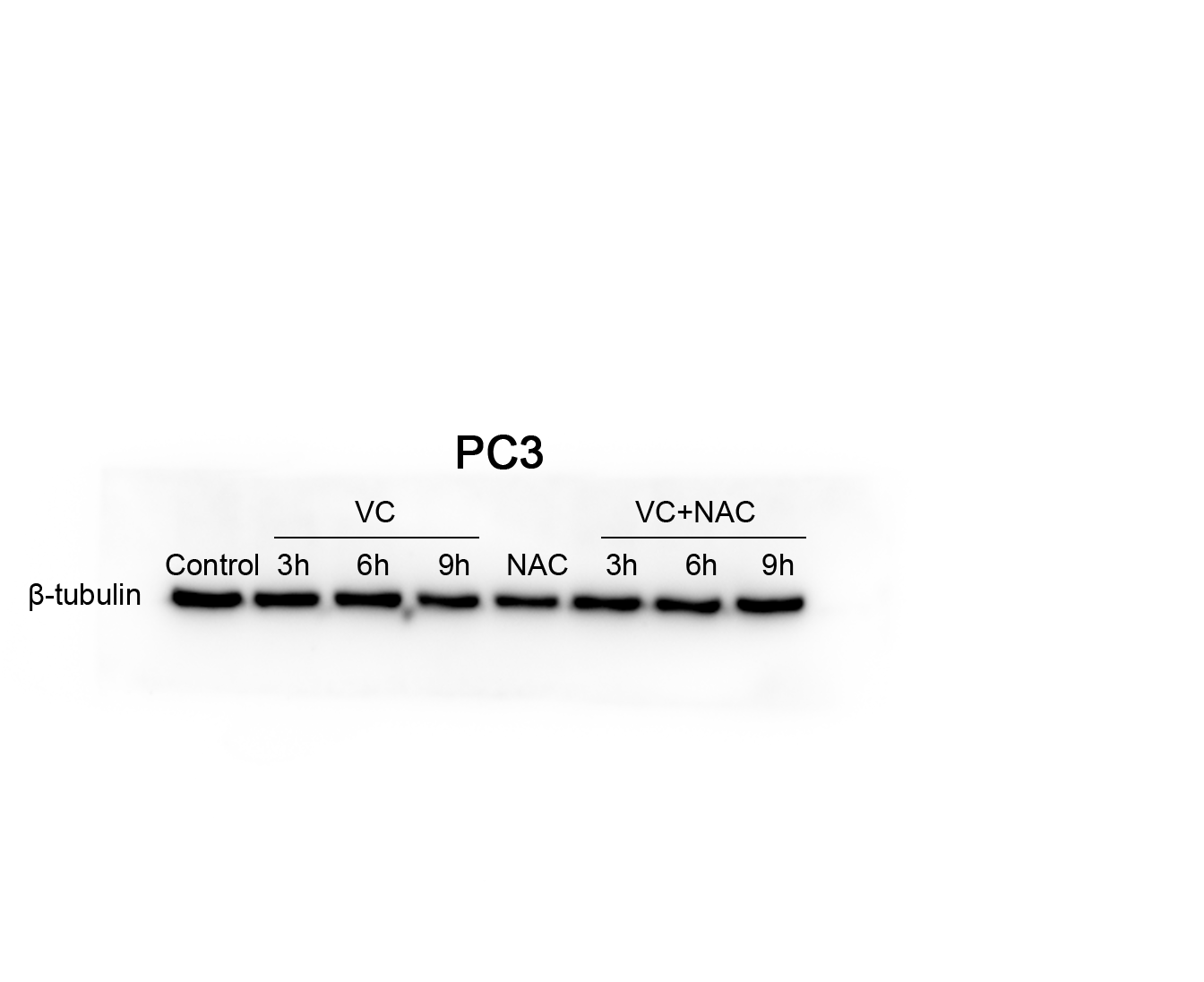

Supplement: Supplementary file 1 [file image6.tif]

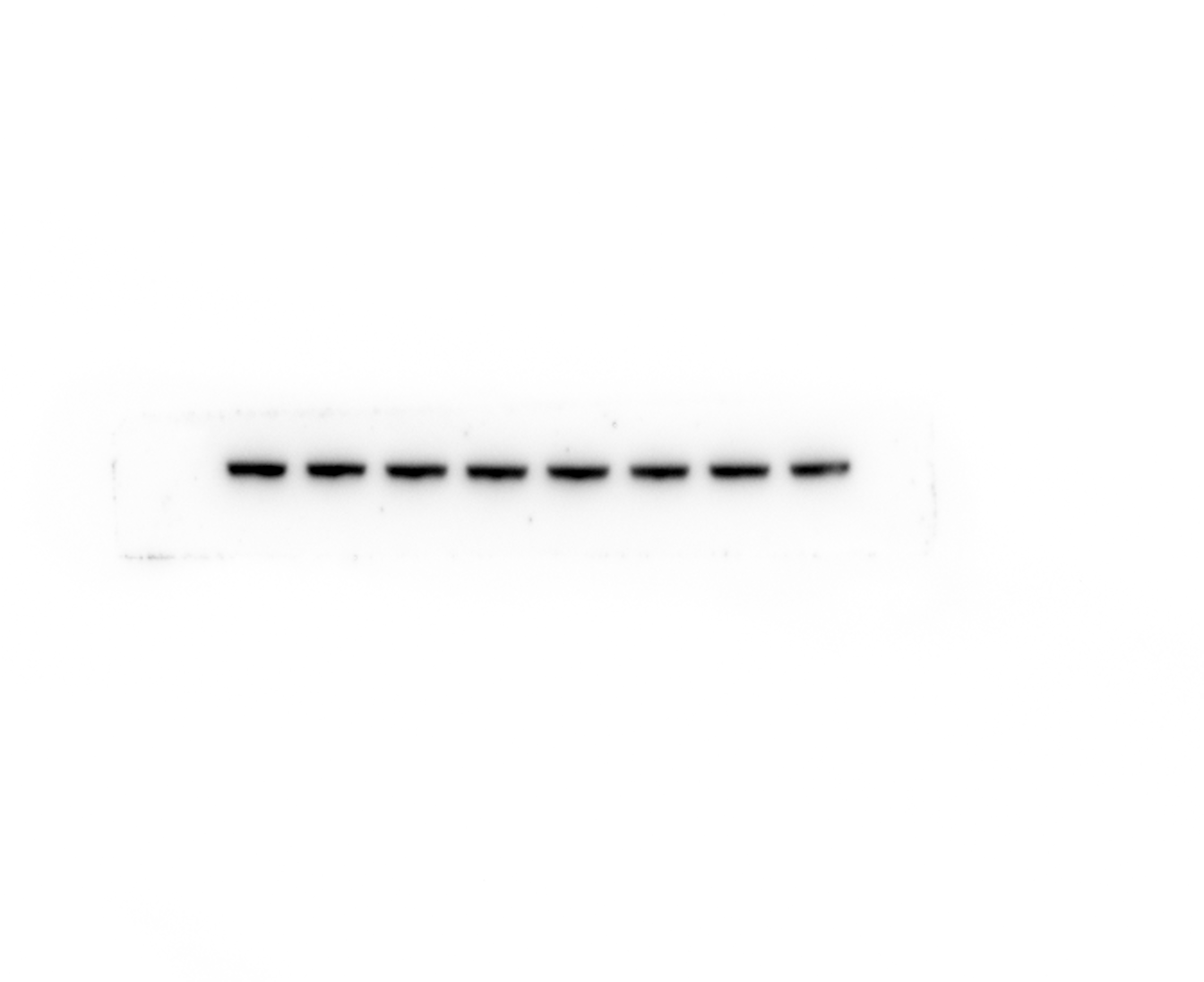

Supplement: Supplementary file 2 [file image14.tif]

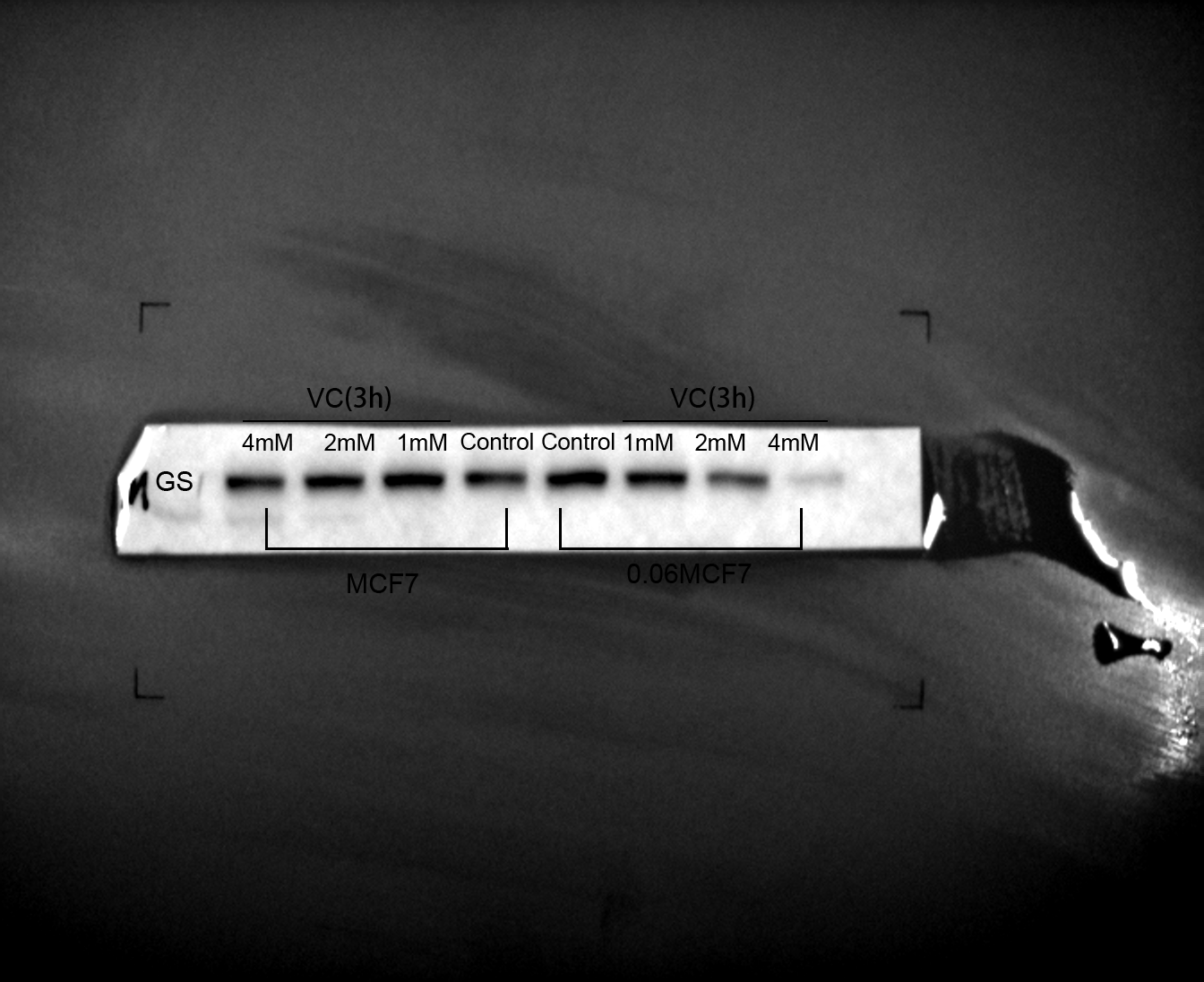

Supplement: Supplementary file 3 [file image3.tif]

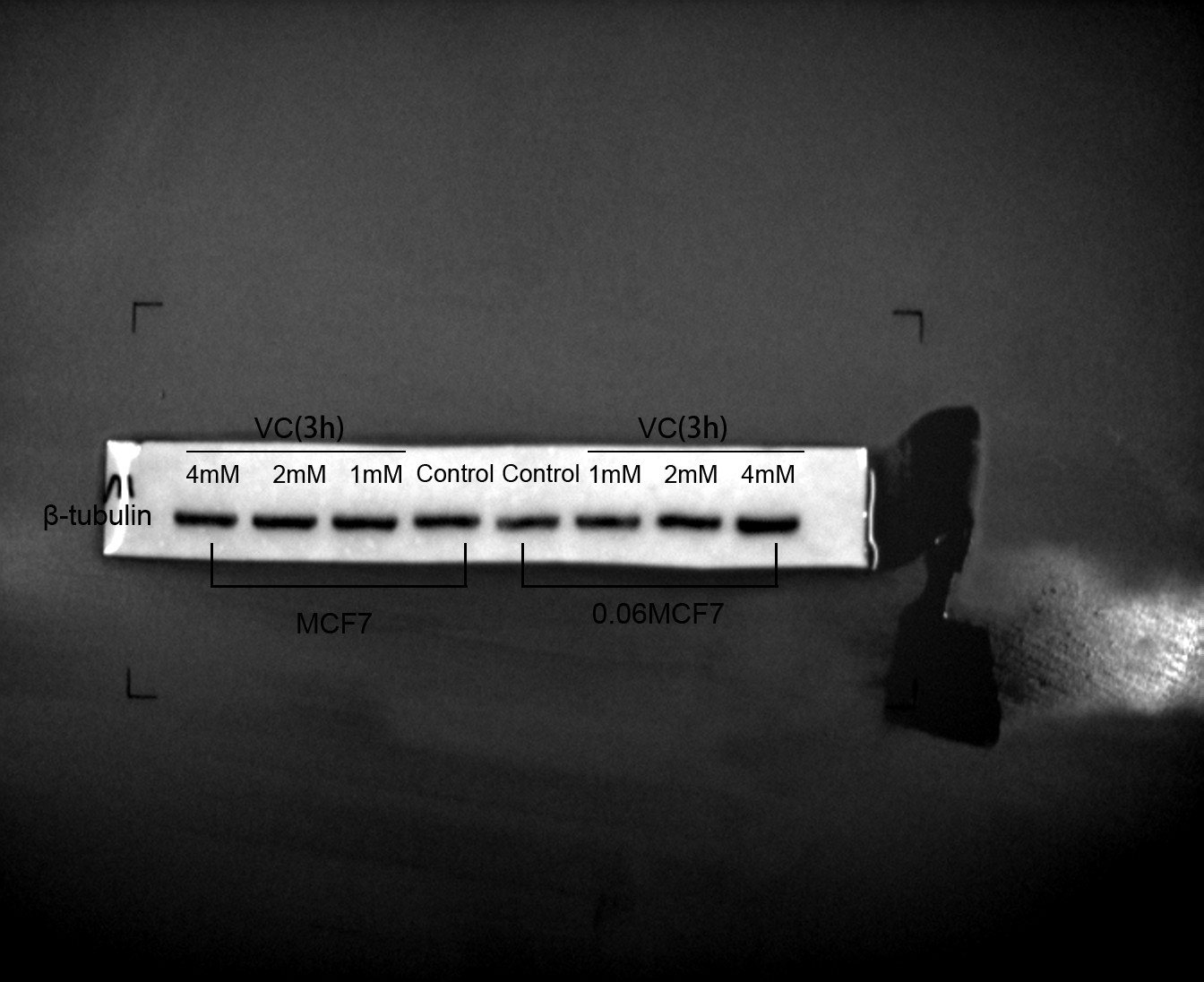

Supplement: Supplementary file 4 [file image4.tif]

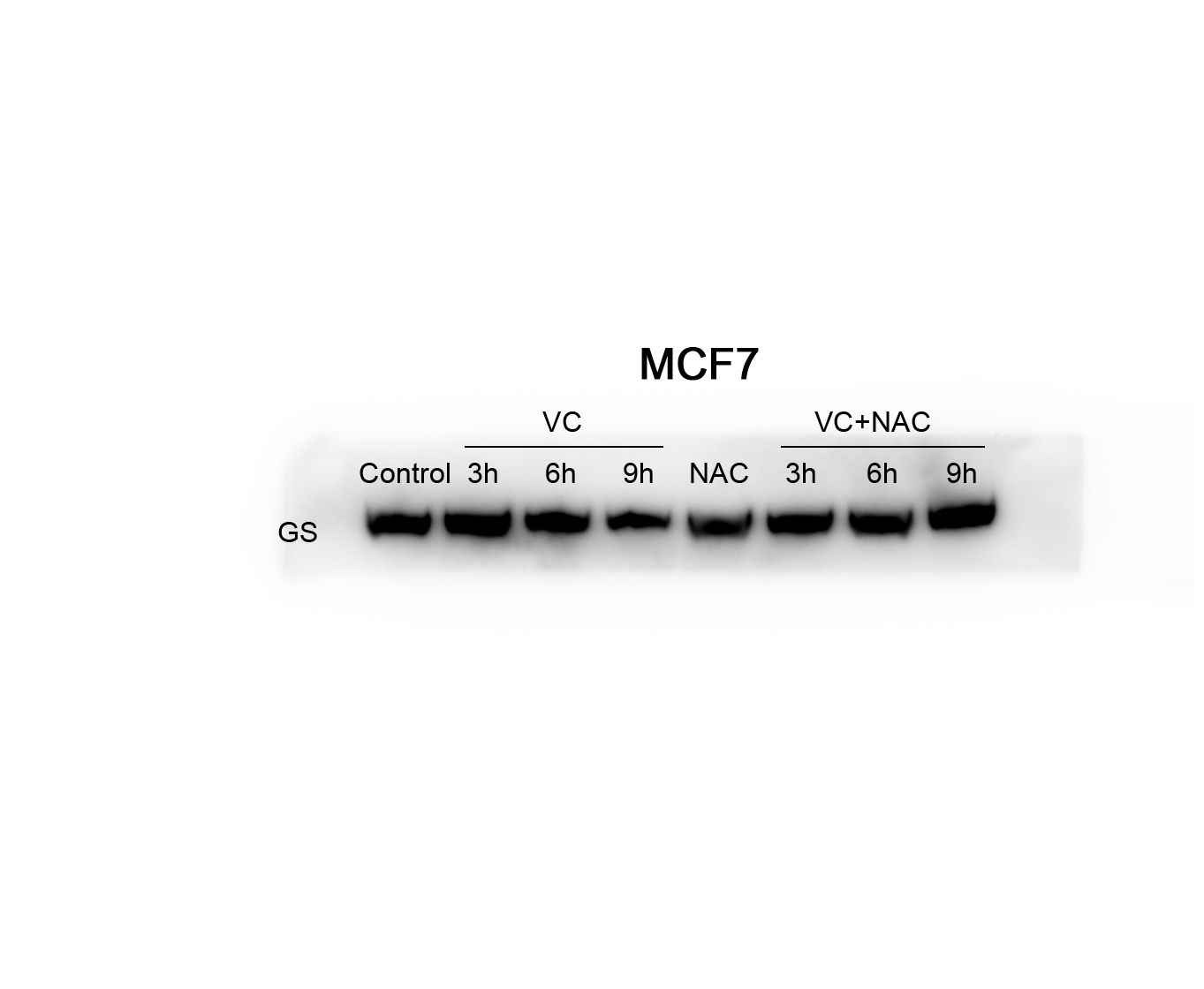

Supplement: Supplementary file 5 [file image9.tif]

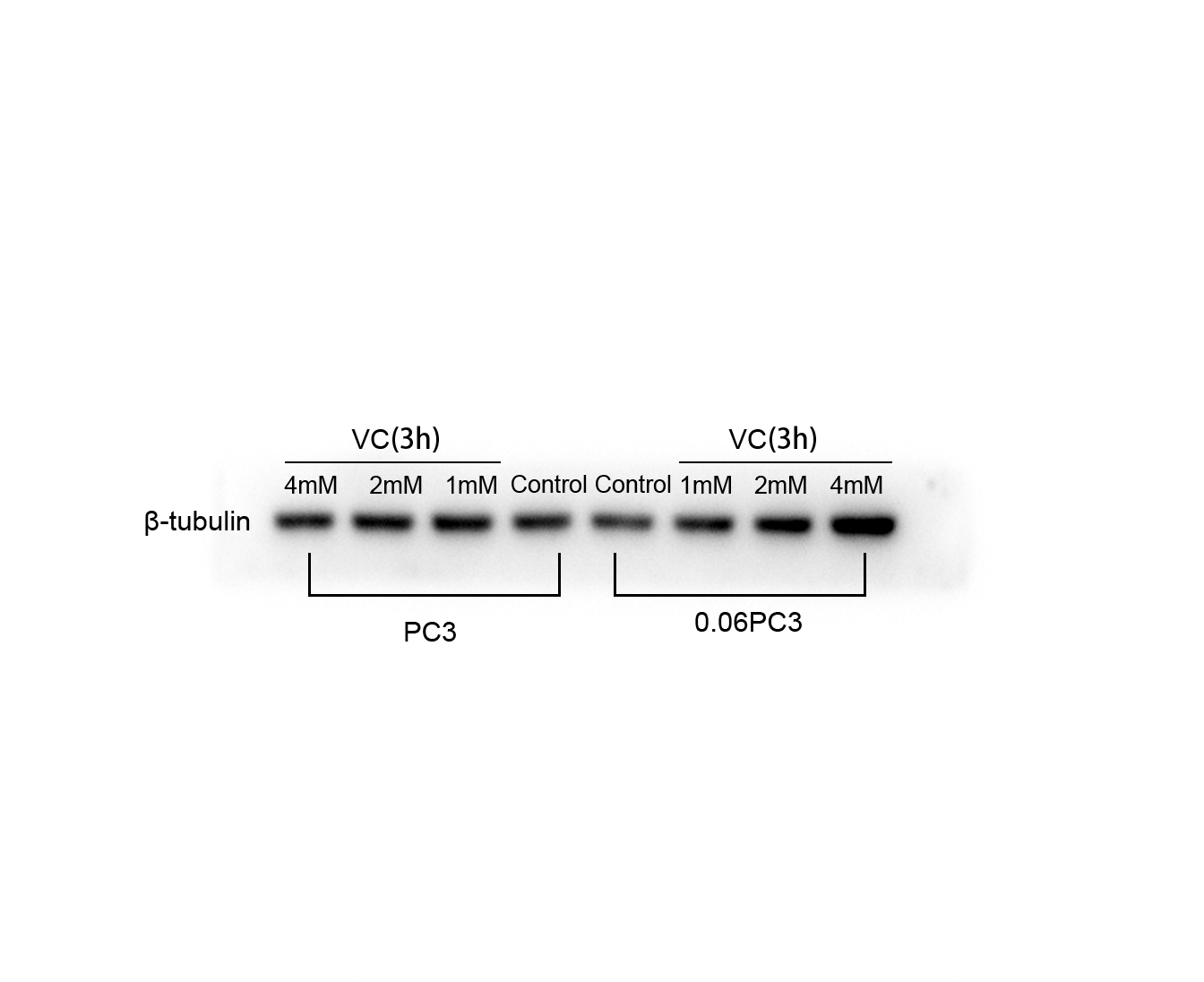

Supplement: Supplementary file 6 [file image2.tif]

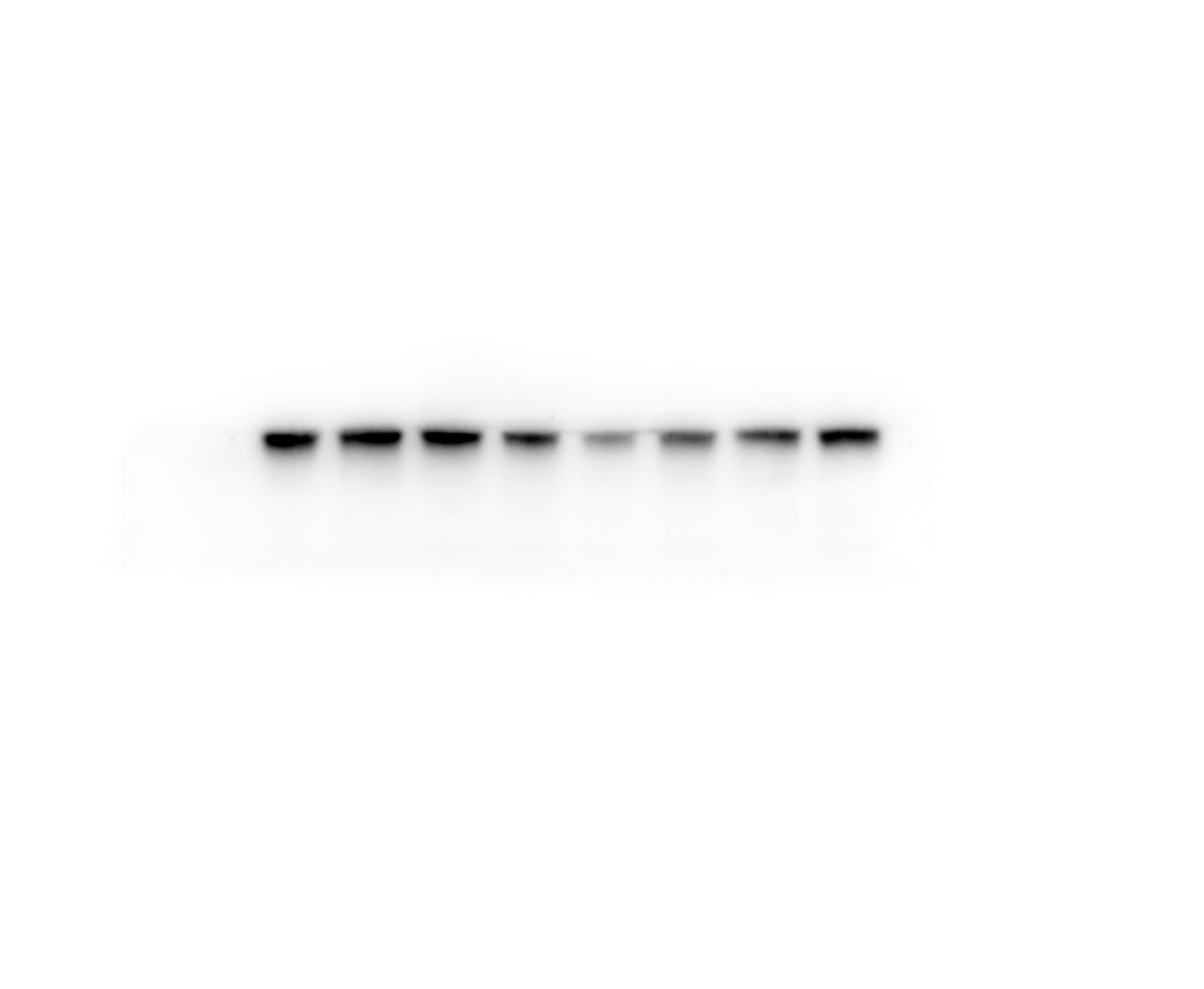

Supplement: Supplementary file 7 [file image13.tif]

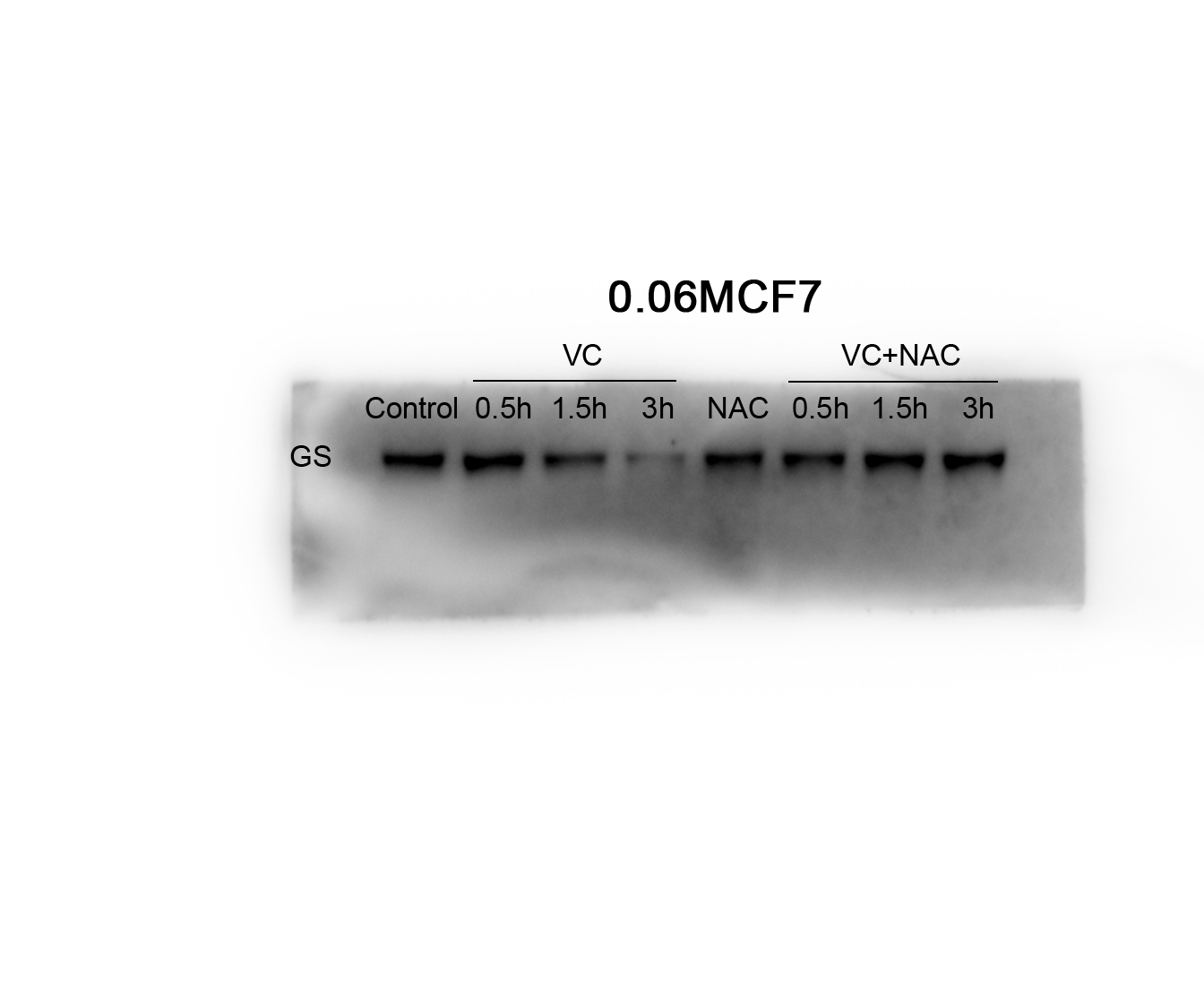

Supplement: Supplementary file 8 [file image11.tif]

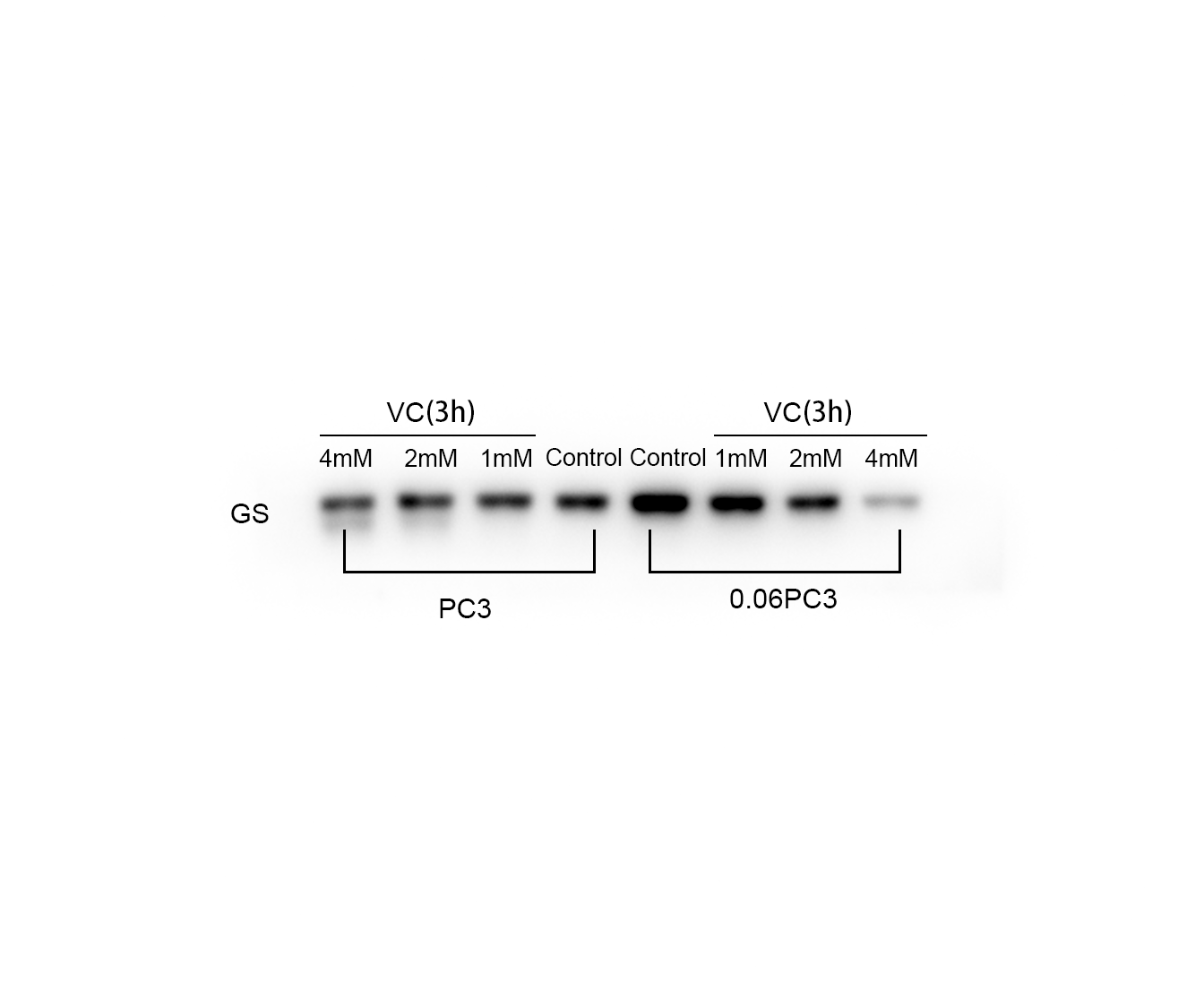

Supplement: Supplementary file 9 [file image1.tif]

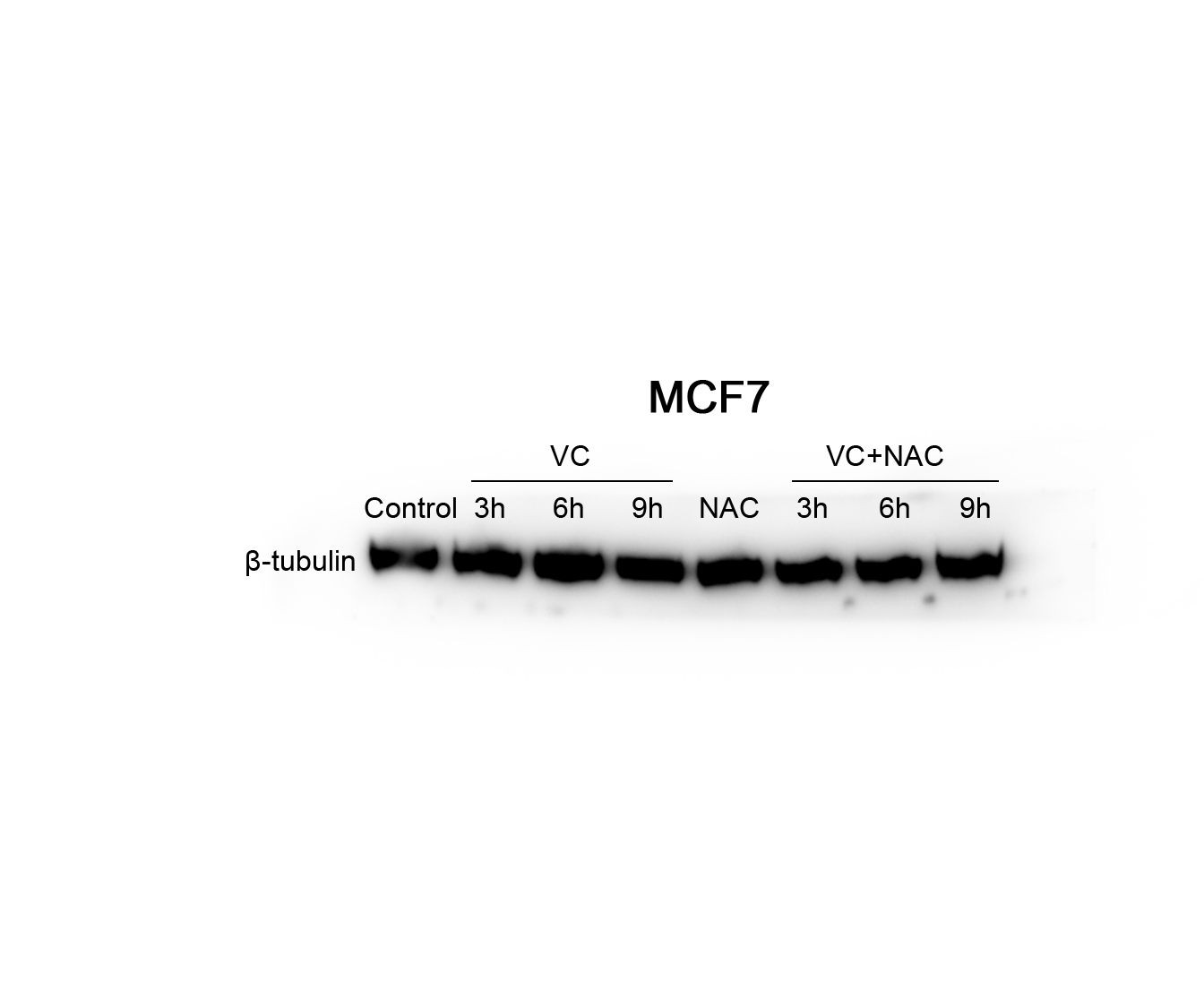

Supplement: Supplementary file 10 [file image10.tif]

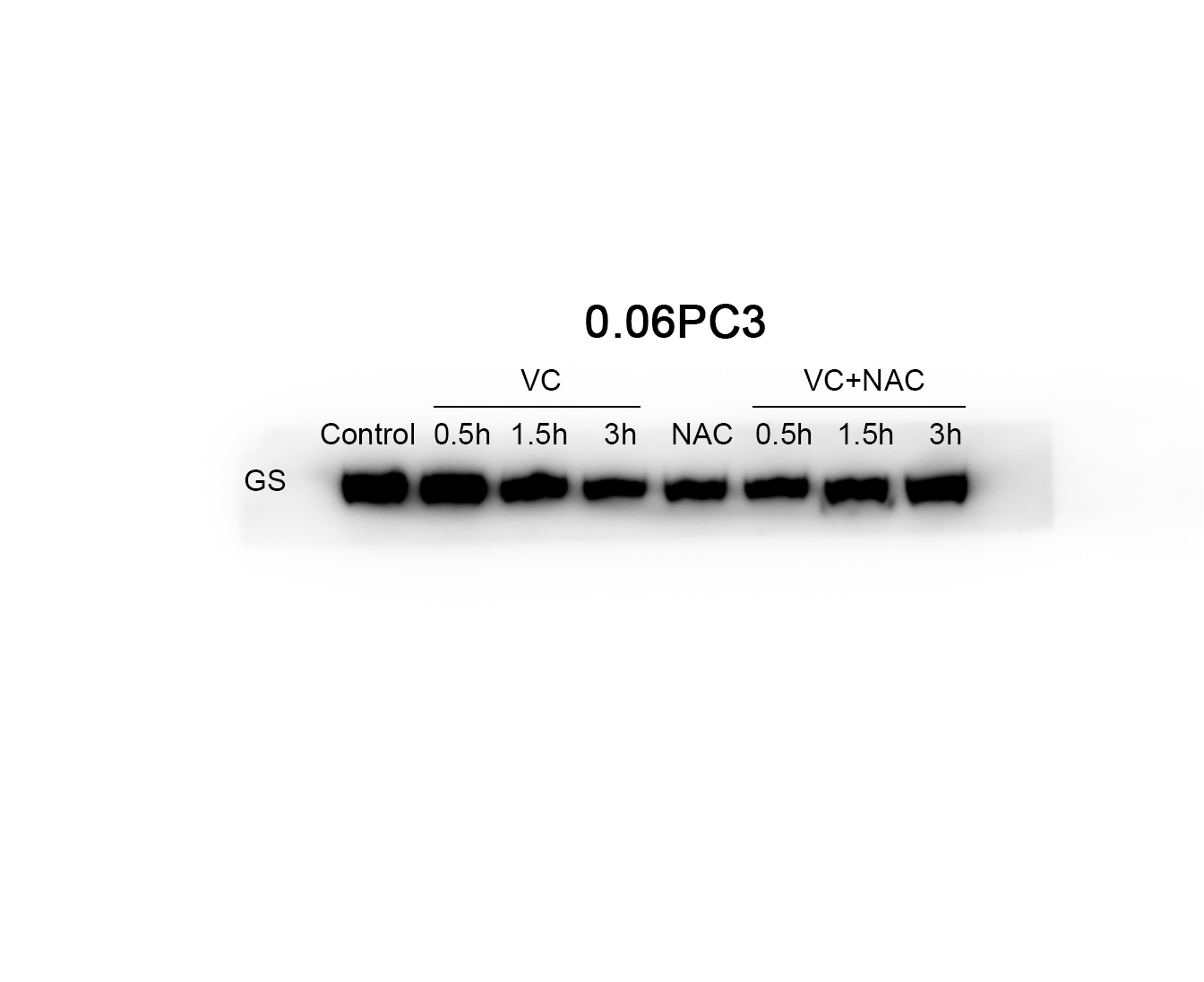

Supplement: Supplementary file 11 [file image7.tif]

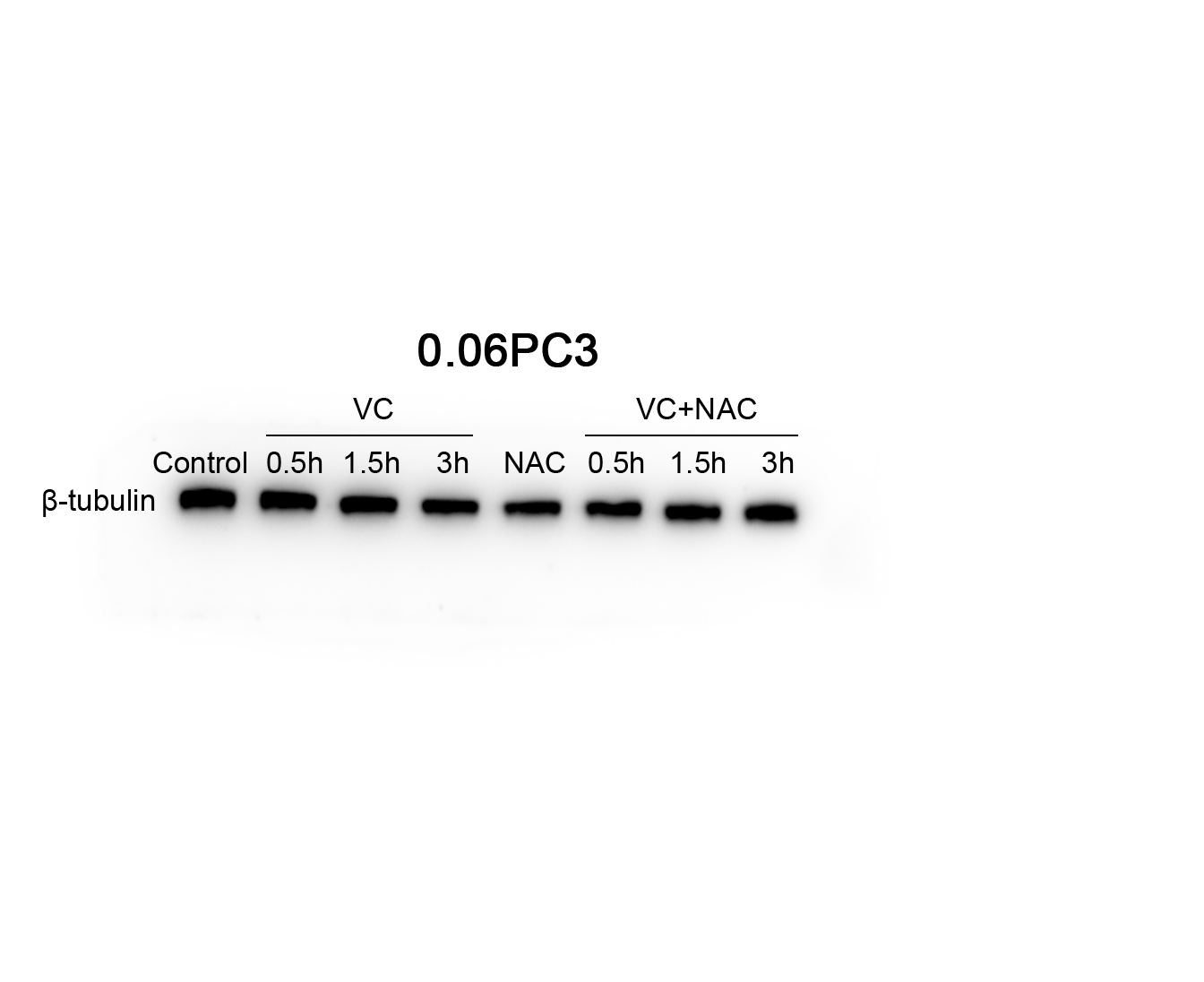

Supplement: Supplementary file 12 [file image8.tif]

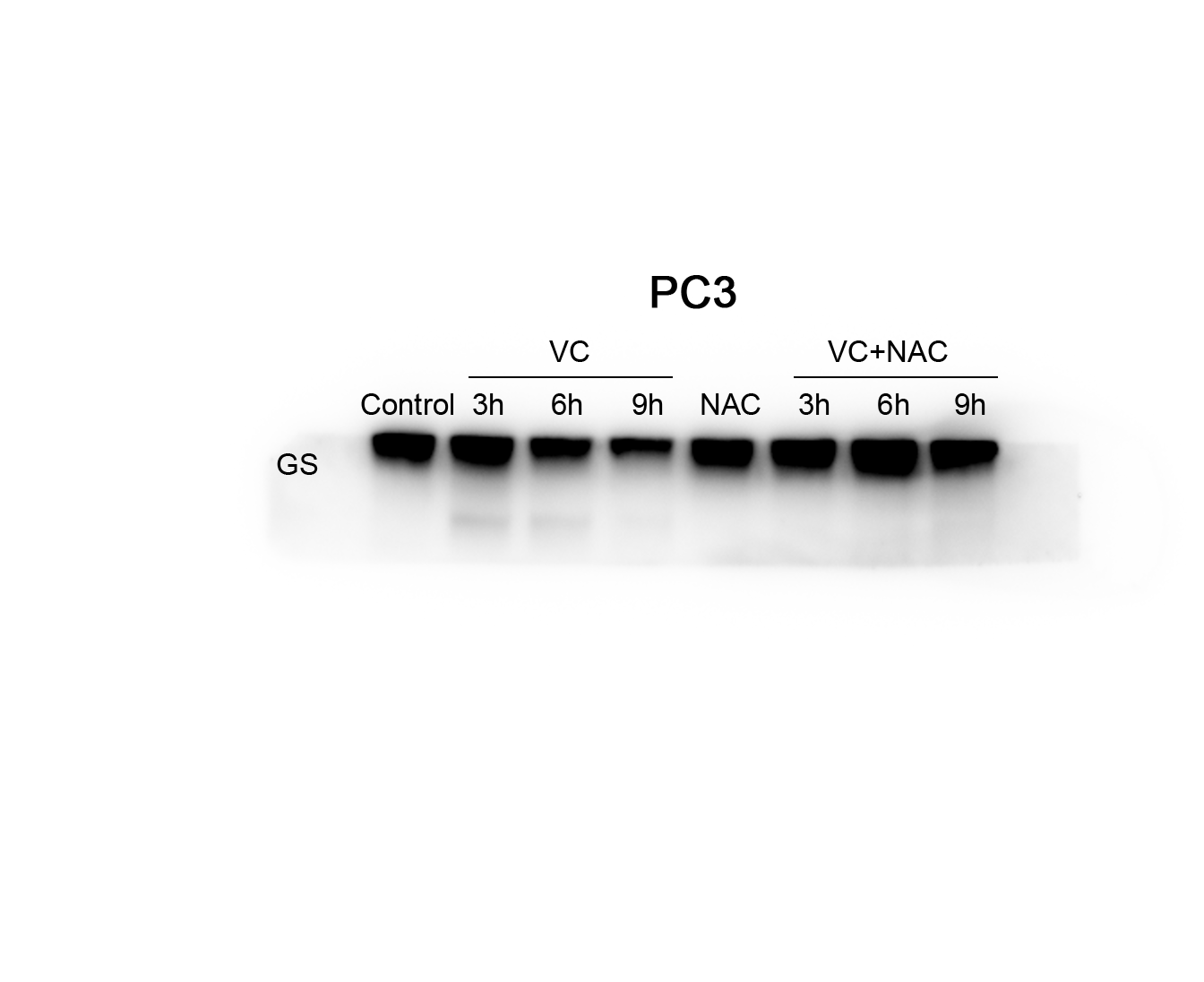

Supplement: Supplementary file 13 [file image5.tif]

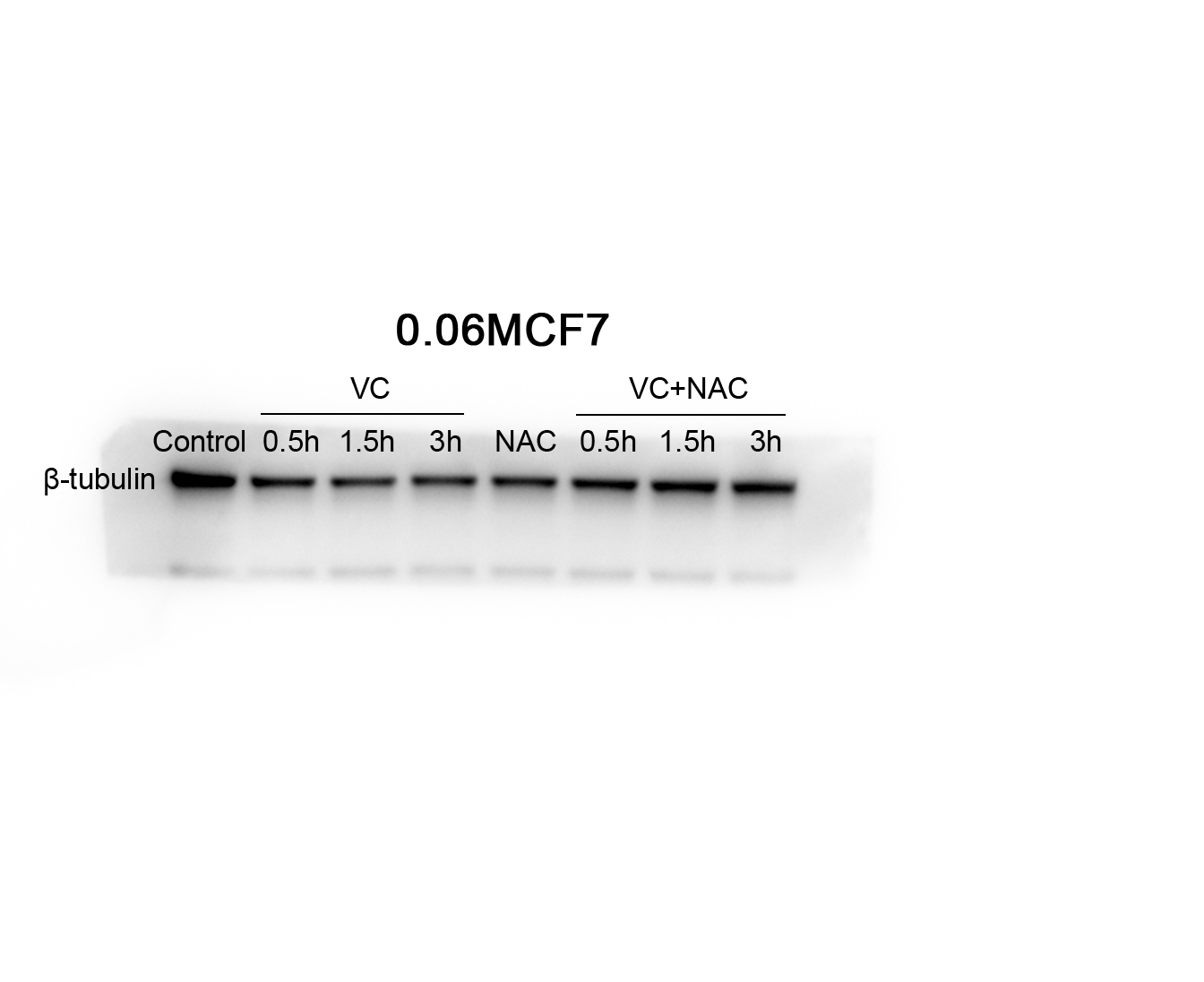

Supplement: Supplementary file 14 [file image12.tif]
